# From Attachment to Damage: Defined Genes of *Candida albicans* Mediate Adhesion, Invasion and Damage during Interaction with Oral Epithelial Cells

**DOI:** 10.1371/journal.pone.0017046

**Published:** 2011-02-23

**Authors:** Betty Wächtler, Duncan Wilson, Katja Haedicke, Frederic Dalle, Bernhard Hube

**Affiliations:** 1 Department of Microbial Pathogenicity Mechanisms, Leibniz Institute for Natural Product Research and Infection Biology – Hans Knoell Institute Jena (HKI), Jena, Germany; 2 Laboratoire Interaction Muqueuses-Agents transmissibles (EA 562), IFR Santé-STIC, Université de Bourgogne, Faculté de Médecine, Dijon, France; 3 University Hospital, Dijon, France; 4 Friedrich Schiller University, Jena, Germany; University of Aberdeen, United Kingdom

## Abstract

*Candida albicans* frequently causes superficial infections by invading and damaging epithelial cells, but may also cause systemic infections by penetrating through epithelial barriers. *C. albicans* is an unusual pathogen because it can invade epithelial cells via two distinct mechanisms: induced endocytosis, analogous to facultative intracellular enteropathogenic bacteria, and active penetration, similar to plant pathogenic fungi. Here we investigated the molecular basis of *C. albicans* epithelial interactions. By systematically assessing the contributions of defined fungal pathways and factors to different stages of epithelial interactions, we provide an expansive portrait of the processes and activities involved in epithelial infection. We strengthen the concept that hyphal formation is critical for epithelial invasion. Importantly, our data support a model whereby initial epithelial invasion per se does not elicit host damage, but that *C. albicans* relies on a combination of contact-sensing, directed hyphal extension, active penetration and the expression of novel pathogenicity factors for further inter-epithelial invasion, dissemination and ultimate damage of host cells. Finally, we explore the transcriptional landscape of *C. albicans* during the early stages of epithelial interaction, and, via genetic analysis, identify *ICL1* and *PGA34* as novel oral epithelial pathogenicity factors.

## Introduction

The yeast *Candida albicans* is both a harmless commensal and an aggressive pathogen. Depending on the anatomical niche in question, up to 70% or more of the population are colonized with *C. albicans* without any sign of disease [1], [2], [3]. The normal bacterial flora of mucosal surfaces, physical barriers, such as epithelial layers, and a functional immune system maintain the commensal phase of *C. albicans* colonization. However, *C. albicans* frequently overgrows the microbial flora and causes superficial infections and epithelial damage [1], [4]. In severe cases, the fungus can penetrate through epithelial layers into deeper tissues, reach the blood stream and, from there, may cause life-threatening systemic infections. How the transition from a harmless commensal to an aggressive pathogen is triggered is still unknown [5]. Clearly, adhesion to epithelial cells is a key event in both the commensal and pathogenic lifestyles of *C. albicans* [6], [7], [8]. However, the molecular mechanisms by which *C. albicans* attaches to epithelial surfaces, invades various epithelial barriers, causes damage or disseminates within the host are only partially understood [9], [10], although, it has recently been shown that *C. albicans* can gain entry to host epithelial cells via two distinct invasion mechanism: induced endocytosis and active penetration [10]. However, invasion into enterocytes occurs via active penetration only, indicating that epithelial cells differ in their susceptibility to the fungus [10].

One of the best studied virulence attributes and characteristics of *C. albicans* is the ability to change morphologies from yeast-to-hyphal growth (dimorphism) in response to environmental changes. However, the transcriptional programmes associated with dimorphism are also critical for virulence and it is often difficult to disentwine the contribution to pathogenicity of morphology and genes expressed during the different growth forms, since regulators of morphology also influence the expression of other virulence factors [11], [12], [13].

In addition to dimorphism, a number of fungal attributes, such as the expression of adhesion factors, directed growth/thigmotropism, stress adaptation, metabolic flexibility and the secretion of hydrolytic enzymes are implicated in the infection process. Relevant genes which contribute to these infection-associated processes are summarized in Table S1.

We hypothesized that different fungal processes and activities (Table S1) may play different roles during distinct stages of oral candidosis. In this study we therefore undertook a systematic approach to examine the contributions of these different fungal activities to oral epithelial infection. We selected a set of 26 mutants lacking factors that we hypothesized to be important for epithelial invasion (including signaling components, adhesion factors, vacuole biogenesis, intracellular glycerol accumulation and some genes with previously described roles in infection – Table S1) and assessed their ability to adhere to, invade and damage oral epithelial cells. To strengthen this systematic analysis, we performed genome-wide transcriptional profiling of *C. albicans* infecting oral epithelial monolayers and went on to functionally characterize genes identified as up-regulated during epithelial infection.

Importantly, we identified fungal genes and activities which are necessary for distinct stages of *C. albicans* interacting with epithelial cells, including a subset of genes, such as *EED1* and *PGA34*, which are dispensable for epithelial invasion but essential for damage of epithelial cells.

## Results

### Defined *C. albicans* pathways, processes and activities: diverse contributions to epithelial adhesion, invasion and damage

To explore the molecular basis of *C. albicans*-epithelial interactions, we adopted a systematic approach to define the molecular mechanisms underpinning *C. albicans*-epithelial interactions. A total of 26 mutants were selected, which either were defective in processes or activities which we predicted to be important for infection, or which had previously been described as attenuated during host-pathogen interactions. These functional groups of factors include regulators of morphology and/or gene expression (*RAS1, EED1, EFG1, CPH1, CPH2, TPK1, TPK2, TEC1, TUP1, BCR1, HGC1*, *RIM101 and CZF1*), genes encoding cell surface localized and/or hyphal-associated proteins (*ALS3, HWP1*, *HYR1* and *ECM33*), genes encoding factors involved in glycerol homeostasis, (*GPD2*, *GPP1*, *PMT2*), morphogenetic plasticity (*VPS11, GPP1*), detection of physical contact (*MKC1*), alkaline pH response (*RIM101*), factors involved in maintenance of cell polarity, directed growth, calcium influx and homeostasis (*CKA2*), and/or thigmotropism (*RSR1, BUD2, EED1*) and the gene encoding a phospholipase (*PLB1*) (Table S1). All analyzed mutant strains were homozygote deletion mutants except the *pmt2*Δ/*PMT2* deletion strain, as the homozygote *pmt2Δ*/*pmt2Δ* mutant is not viable [14].

We independently assessed the ability of each mutant to adhere to, invade and cause damage to oral epithelial cells. Furthermore, all mutants were additionally tested for enterocyte invasion capacity. Since invasion into enterocytes is entirely dependent on active penetration and independent of induced endocytosis [10], this additional series of experiments enabled us to conclude which invasion mechanism was predominantly attenuated in these mutants. Similar to our previous study [10], the wild type showed comparable adherence, invasion and damage properties when interacting with oral epithelial cells or enterocytes (data not shown). Contact to epithelial or intestinal cells induced filamentation in almost all yeast cells (>98%) within 60 min and caused epithelial adhesion (>98%) within 3 h. Although the morphological phenotypes of most of the mutants used in this study have been previously published (Table S1) we reasoned that some mutants may behave differently on epithelial cells. We therefore determined the percentage of hyphal, pseudohyphal and yeast cells and length of the formed filaments for each strain following 3 h incubation on epithelial monolayers (Table S2).

To determine adhesion, wild type and mutant cells were adjusted to an inoculum of 1×10^5^ cells, added to and co-incubated with monolayers of oral epithelial cells. Adhesion was quantified after 1 h of incubation. Independently, *C. albicans* cells were incubated on monolayers for 3 h and invasion of adherent cells quantified using a differential staining procedure and by calculating the percentage of invaded cells (note that for the invasion assay, monolayers were only washed at 3 h post-infection). In a third set of experiments, damage was quantified after 24 h infection by monitoring the release of epithelial LDH as a measure of cellular lysis (note that for the damage assay, monolayers were not washed during the 24 h incubation and fungal cells were not removed from the epithelial monolayer during this time). Only mutants with statistically significant differences compared to their corresponding wild type (BWP17+CIp30, CAI4+CIp10, RM1000, or SC5314) of *p<*0.05 were considered as different. Since the phenotypes of all tested parental strains in terms of adhesion, invasion and damage of epithelial cells were similar to the wild type strain SC5314, only the results for strain SC5314 were included in the figures.

Based on these analyses, we divided the mutants into five different functional groups: (1) no significant differences, (2) adhesion, invasion and damage reduced, (3) invasion and damage reduced, (4) damage reduced, (5) adhesion and damage reduced. None of the tested mutants displayed significantly increased adherence, invasion or damage potential.

### Group 1: genes not involved in adhesion, invasion and damage

Few of the selected mutants showed no significant differences in adhesion, invasion or damage as compared to wild type cells. These were null mutants lacking the genes *CPH1, CPH2, PLB1* or *HYR1*, indicating that these genes do not play any significant roles during interaction with epithelial cells under the conditions tested (data not shown).

### Group 2a: genes associated with adhesion, invasion and damage

**Figure 1:**
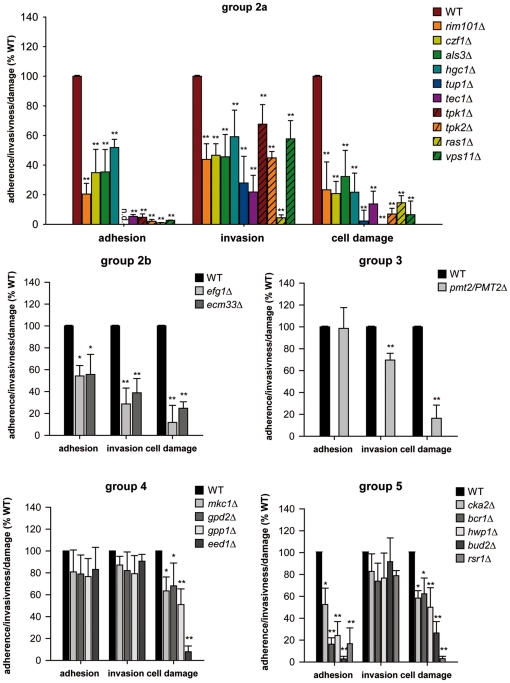
Groups of mutants lacking genes associated with adhesion, invasion and damage. Adhesion, invasion and damage potential was determined for each mutant and normalized against the relevant wild type. Group 2a mutants showed reduced adhesion, invasion and damage. The group 2b mutants *efg1*Δ and *ecm33*Δ show significant reduction in adhesion, but have a further decreased ability to invade into epithelial cells, suggesting distinct adhesion and invasion functions of these genes. The group 3 mutant *pmt2*Δ/*PMT2* lacks factors or activities which are specifically associated with invasion and damage, but not adhesion. Group 4 mutants lacking *MKC1*, *GPP1, GPD2* and *EED1* showed significant reduction in damage, but not adhesion and invasion. Group 5 mutants lacking *CKA2*, *BCR1, HWP1*, *BUD2* or *RSR1* showed significant reduction in adhesion and damage, but not invasion. n. d. =  not determined; */**, significant difference compared to the corresponding wild type (WT) (*p<*0.05/*p<*0.01). Note that adhesion, invasion and damage assays were performed separately.

Null mutants lacking the genes *RIM101*, *CZF1*, *ALS3*, *HGC1*, *TUP1*, *TEC1*, *TPK1*, *TPK2*, *RAS1*, or *VPS11* showed significant differences at all analyzed stages of infection (adhesion, invasion, damage) (Fig. 1). Almost all of these mutants showed dramatically reduced adhesion (less than 35% of the wild type). Most of these mutants retained a certain degree of invasion potential (>40% of the wild type), however, the *ras1*Δ mutant was almost entirely unable to invade epithelial cells. Similar to the reduction in adhesion, damage was strongly reduced for all mutants with *tpk1*Δ showing no measurable damage. Therefore, we concluded that group 2a genes are associated with adhesion, invasion and damage at all stages. All of these mutants also had significantly reduced abilities to invade enterocytes (data not shown, Table S3), suggesting that this group had reduced abilities to actively penetrate host cells. An exceptional member of group 2 was the mutant lacking *TUP1*. This mutant is hyperfilamentous, does not grow in the yeast form, and produces cell clumps, which causes experimental problems since standard cell counting to adjust to an exact defined cell number for infection is impossible. Therefore, we filtered *tup1*Δ cells grown in liquid precultures to remove larger cell clumps, counted cells under the microscope and adjusted the inoculum to approximately 2×10^4^ cells. Since exact cell numbers are essential for quantification of adhesion, we could not quantify adhesion for this mutant. However, since the invasion assay is based on percent invasion of cells on epithelial surfaces, quantification of invasion was possible for *tup1*Δ. Quantification of cell damage also depends on exact cell numbers, however, since the damage of *tup1*Δ was barely measurable (less than 5% compared to the wild type), which cannot be explained by moderately lower cell numbers, we concluded that damage of *tup1*Δ is strongly reduced.

### Group 2b: genes associated with adhesion, invasion and damage; adhesion-independent functions in invasion

Group 2b comprised *efg1*Δ and *ecm33*Δ and showed reduced adhesion, reduced invasion and reduced damage. Therefore, these genes belong to group 2. However, in contrast to group 2a, the invasion attenuation of group 2b mutants was much stronger than the observed reduction in adhesion (Fig. 1). Adhesion of *efg1*Δ was 45% reduced, but invasion of *efg1*Δ into epithelial cells was more than 70% reduced. Similarly, adhesion of *ecm33*Δ was 45% reduced, but invasion of *ecm33*Δ into epithelial cells was more than 60% reduced, suggesting that *EFG1* and *ECM33* play additional adhesion-independent roles in invasion. Furthermore, invasion of *efg1*Δ into enterocytes was dramatically reduced (less than 5% of wild type,) while invasion of *ecm33*Δ into enterocytes was reduced by 50% (data not shown), indicating that both mutants had reduced abilities to actively penetrate host cells.

Interestingly, all tested mutants with defects in the cAMP-PKA-Efg1 signaling pathway (*ras1*Δ, *tpk1*Δ, *tpk2*Δ, *efg1*Δ, *tec1*Δ) belonged to group 2, or the related sub-group 2b and had defects in adhesion, invasion and damage.

### Group 3: *PMT2* plays a specific role in epithelial invasion and damage

Interestingly, of all strains tested, only one mutant (*pmt2*Δ/*PMT2*) exhibited unaltered adhesion properties but significantly reduced invasion (>30% reduced) and damage capacity (>80% reduced) (Fig. 1). Similarly, invasion into enterocytes was strongly reduced (less than 30% of wild type, data not shown), suggesting that *pmt2*Δ/*PMT2* is defective for active penetration of host cells.

### Group 4: genes which are specifically associated with damage

In this group, neither adhesion nor invasion of mutants was significantly altered, but damage of oral epithelial cells was significantly reduced (Fig. 1). The mutants in this group include strains lacking the genes *MKC1, GPD2*, *GPP1* or *EED1*. In particular, damage of oral epithelial cells by *eed1*Δ was dramatically reduced. An *EED1* re-integrant strain restored wild type damage (data not shown). Interestingly, all damage associated strains of this group were able to form hyphae comparable to the wild type strain SC5314 by 3 h post-infection. Therefore, these mutants lack genes, which are specifically involved in damage of oral cells, but not adhesion or invasion. Since both *GPD2* and *GPP1* are involved in glycerol metabolism, we hypothesized that reduced intracellular glycerol content and resulting turgor pressure may have influenced the damage potential of mutant cells. We therefore measured the glycerol content of *gpp1*Δ and *gpd2*Δ mutants under osmotic stress conditions. Only the *gpp1*Δ mutant contained significantly less glycerol –36.1±2.5% (*p<*0.0017) as compared to the level of wild type cells – under this condition.

### Group 5: genes which are specifically associated with adhesion and damage, but not invasion

Null mutants lacking the genes *CKA2*, *BCR1*, *HWP1*, *BUD2* or *RSR1* were strongly reduced in adhesion (at 1 h), but not significantly reduced in invasion into oral epithelial cells (at 3 h) (Fig. 1). Despite normal invasion potential, damage of oral monolayers was, like group 4 mutants, significantly reduced. Therefore, these genes seem to play a role in initial adhesion and subsequent damage, but are dispensable for invasion at 3 h. Together with the data for group 4, it would appear that considerable (i.e. wild type) levels of epithelial invasion can occur without eliciting significant damage of the host cells, suggesting that although oral epithelial invasion is required for damage, invasion *per se* does not cause damage and that other fungal activities are involved in epithelial destruction.

### Group 4 and 5: stage specific roles in epithelial infection

Group 4 and 5 mutants displayed specific defects during epithelial infection. Of particular interest was the fact that these mutants invaded at the same rate as the wild type, but caused significantly reduced epithelial damage. We therefore decided to further dissect the roles of these “damage-associated” group 4 and 5 genes during epithelial infection.

### Differential adhesion kinetics (group 5)

**Figure 2:**
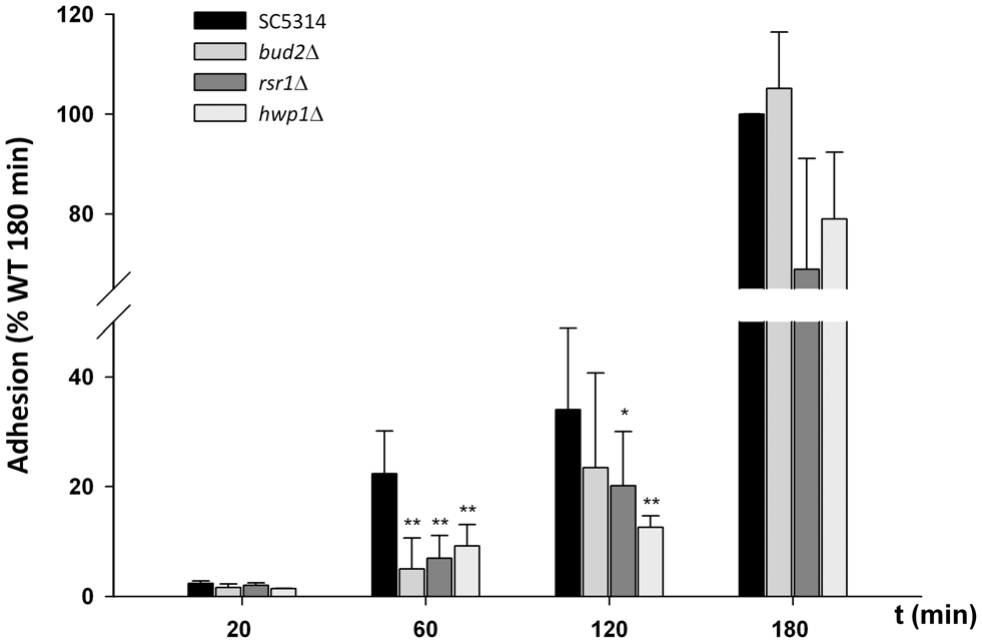
Adhesion kinetics of *C. albicans* wild type (WT), *bud2*Δ, *rsr1*Δ and *hwp1*Δ mutant cells. Oral TR146 epithelial cells were co-incubated with 10^5^ *C. albicans* cells for either 20, 60, 120, or 180 min. After extensive washing and fixation, the samples were stained and the adherent cells were counted under the fluorescence microscope. The experiment was performed at least three times in duplicates. The values are calculated as percentage of adherent cells compared to wild type adherent cells at 180 min (100%). */**, significant difference compared to the adhesion of the corresponding WT to oral epithelial cells (*p<*0.05/*p<*0.01).

The reduced initial adhesion (1 h) but unattenuated invasion (3 h) of group 5 mutants suggested that the respective gene products had major influences on initial adhesion, but that their requirements are then bypassed following extended epithelial contact. We therefore analyzed the adhesion kinetics of wild type *C. albicans*, as well as mutants lacking the regulatory GTPase Rsr1 (encoded by *RSR1*), involved in maintenance of cell polarity, directed growth and thigmotropism, its cognate GTPase activating protein (encoded by *BUD2*) and the adhesin-encoding *HWP1*. As shown in Fig. 2, adhesion rates at 20 min were low for all tested strains; however, following 1 h incubation, wild type cells began to adhere in greater numbers whilst adhesion rates of *bud2*Δ, *rsr1*Δ and *hwp1*Δ remained low. At 2 h, wild type adhesion had continued to rise and *bud2*Δ and *rsr1*Δ strains adhered in greater numbers; however adhesion of *hwp1*Δ at this time point remained low. Only by 3 h had all strains reached approximately maximum adhesion similar to the wild type (Fig. 2). Therefore, the reduced damage caused by these mutants cannot be explained by fewer initially invading cells. Together these data suggest that primary adhesion of *C. albicans* to epithelium relies on a combination of factors, including GTPase signaling and the expression of appropriate adhesins, but that following prolonged contact (3 h), defects elicited by the deletion of a single gene are compensated for.

### Specific invasion roles (group 4 and 5)

**Figure 3:**
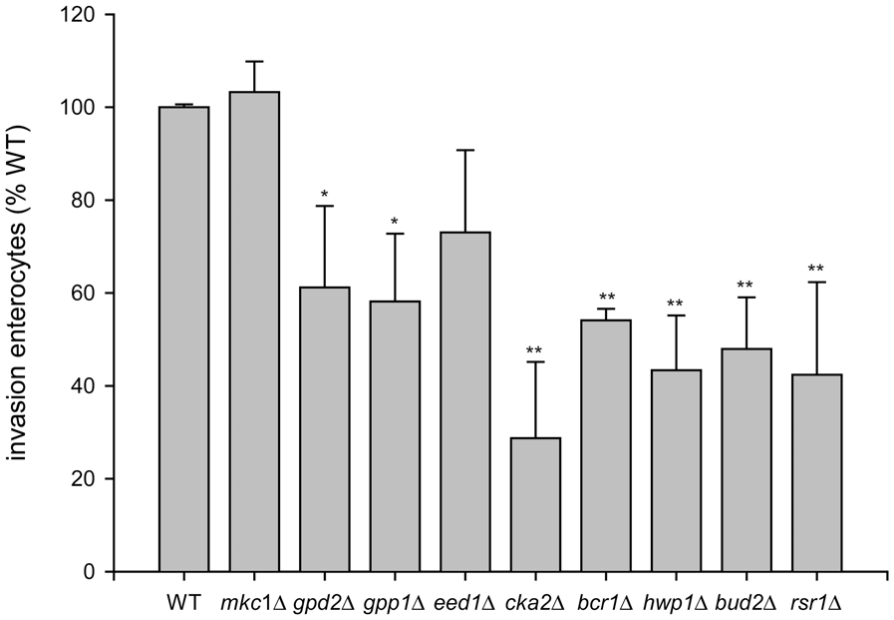
Enterocyte invasion by group 4 and 5 damage-associated mutants. *C. albicans* cells (wild type and *rsr1*Δ, *bud2*Δ, *hwp1*Δ, *bcr1*Δ, *cka2*Δ, *eed1*Δ, *gpp1*Δ, *gpd2*Δ and *mkc1*Δ) were co-incubated with Caco-2 enterocytes for 3 h. After fixation the samples were differentially stained and analyzed under the fluorescence microscope. The experiment was performed at least three times in duplicates. */**, significant difference compared to the corresponding WT invasion (control) (*p<*0.05/*p<*0.01).

Although fully proficient for invasion into TR-146 oral epithelial cells, when tested for invasion into enterocytes, some mutants displayed substantial invasion defects. Indeed, of the nine damage-associated mutants (group 4/5), only two (*eed1*Δ and *mkc1*Δ) exhibited wild type levels of invasion into enterocytes (Fig. 3), suggesting that *EED1* and *MKC1* are not required for invasion into either cell type. On the other hand, *gpd2*Δ, *gpp1*Δ, *cka2*Δ, *bcr1*Δ, *hwp1*Δ, *bud2*Δ and *rsr1*Δ, all of which were fully proficient for oral epithelial invasion (Fig. 1), exhibited strong and significantly reduced invasion into enterocytes (Fig. 3). Together these data show that certain *C. albicans* genes are specifically required for invasion into enterocytes but not oral epithelial cells.

### Damage of oral epithelial multilayers (group 4 and 5)

Because many of the damage-associated genes (group 4/5) are involved in thigmotropism and/or hyphal orientation (Table S1), we next questioned whether they are required for interactions with oral epithelial multilayers, where inter-epithelial dissemination of the wild type predominantly occurs vertically [15], rather than laterally, as is the case for epithelial monolayers.

Wild type and damage-associated mutants (group 4/5) were therefore used to infect reconstituted human oral epithelium (RHOE) and damage assayed following 24 h of infection by measuring LDH release. *eed1*Δ and *hwp1*Δ exhibited significantly reduced damage of RHOE (Table 1). As shown by Zakikhany *et al*., (2007) an *EED1* re-integrant strain completely restored wild type morphology and epithelial damage in the RHOE model [15]. *bcr1*Δ also exhibited a notable reduction in RHOE damage, however this was not statistically significant. Interestingly, *bud2*Δ, *cka2*Δ, *gpd2*Δ, *gpp1*Δ and *mkc1*Δ were not attenuated for damage. The *rsr1*Δ mutant displayed some (37%, non-significant) reduction in RHOE damage; however this was a very moderate attenuation in damage of RHOE in comparison to the strong damage attenuation of *rsr1*Δ associated with epithelial monolayers (97%, Fig. 1). We therefore conclude that *RSR1* plays a substantial role in epithelial monolayer, but not multilayer damage.

**Table 1:** Damage of RHOE tissue caused by wild type and mutant strains after 24 h.

tested mutant strain	LDH release (% WT)
WT (SC5314)	100%
*bcr1*Δ	56.2±8.8
*bud2*Δ	74.7±10.2
*cka2*Δ	77.3±6.1
*ipf946Δ* (*eed1*Δ)	42.4±22.9*
*gpd2*Δ	79.5±32.1
*gpp1*Δ	74.5±21.5
*hwp1*Δ	90.6±0.9*
*mkc1*Δ	88.6±0.5
*pmt2*Δ/*PMT2*	57.2±36.1
*rsr1*Δ	63.0±18.9

Extracellular LDH release was measured as a marker for tissue damage and values are listed as percentage of wild type damage.

*p<0.05 compared to the wild type.

Together these data demonstrate that *BUD2*, *RSR1*, *CKA2*, *MKC1*, *GPD2* and *GPP1* are specifically required for damage of oral epithelial monolayers, but dispensable for damage of multilayers of the same cell type.

In summary, we have demonstrated that certain *C. albicans* genes, such as *EFG1* and components of its upstream regulatory pathway are essential for all stages of *C. albicans*-epithelial interaction, whilst other genes can have discreet functions at different stages of infection. For example, *EED1* is dispensable for invasion into both oral epithelial cells and enterocytes, but required for epithelial damage. On the other hand, genes involved in glycerol homeostasis (*GPD2* and *GPP1*) and directed hyphal growth (*BUD2* and *RSR1*), are specifically required for enterocyte, but not oral epithelial, invasion, and are essential for full epithelial damage.

### Transcriptional profiling during adhesion, invasion and damage of monolayers

Following on from our systematic analysis of known fungal factors, we next analyzed *C. albicans* gene expression during oral infection. Our transcriptional analysis had three major aims. (1) To verify our functional analysis (above): for example, we predicted that certain genes required for epithelial interactions (or, in the case of transcriptional regulators, their target genes) would be up-regulated during infection. (2) To provide further data of the cellular activities of *C. albicans* in response to contact with host cells and host cell activities. (3) To identify novel infection-associated genes and processes.

To analyze the expression profile of *C. albicans* during the early stages of epithelial interaction and invasion, we co-incubated *C. albicans* cells (from exponential phase yeast preculture conditions) with monolayers of oral epithelial cells and isolated RNA from samples at 20, 60 and 180 min for genome-wide transcriptional profiling. We reasoned that analysis of transcriptional changes at these early time points may reflect the fungal activities directly involved in adhesion and invasion, but may also identify genes that are subsequently involved in epithelial damage. Next, the sample RNA was co-hybridized against RNA from the exponential preculture condition (common reference, see *Material and Methods*) to determine temporal fluctuations in gene expression. In parallel, we incubated *C. albicans* cells under the same conditions on the plastic surfaces of cell culture wells but without epithelial cells to identify gene expression responses specific to epithelial cells. Both data sets, differentially regulated genes compared to YPD and to plastic, can be accessed in Table S4 and S5, respectively.

To verify our microarray data, we tested the expression of selected genes (*CRP1*, *OPT9*, orf19.6835, *PGA37*, *GPP1*, orf19.3600, *ECE1* and *ALS3*) by quantitative RT-PCR. Overall, the change in expression of all genes tested by qRT-PCR was in agreement with the direction of fold change as determined by microarray analysis (data not shown).

### Overview of gene expression in response to oral cells

**Figure 4:**
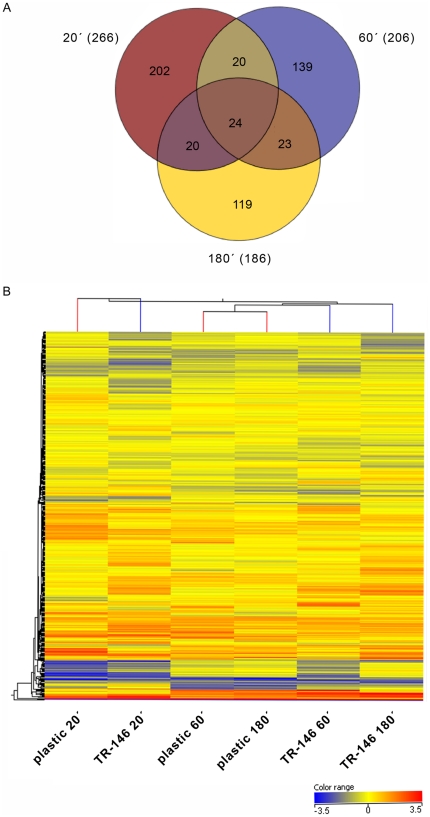
Distribution of differentially regulated genes and hierarchical clustering. (A) Venn diagram showing the distribution of the differentially regulated genes from *C. albicans* cells grown on epithelial monolayer at 20, 60 and 180 min after horizontal analysis as compared to plastic. (B) Hierarchical clustering of *C. albicans* genes expressed at 20, 60 and 180 min incubation on oral epithelial cells versus plastic. Gene expression is shown in a logarithmic color range from −11.31 in blue to 11.31 in red. Non regulated genes (1) are shown in yellow. The profiles of the earliest time points cluster more distant to the later time points. The expression profiles of *C. albicans* exposed to epithelial cells at 20, 60 and 180 min did not show any clear similarities. In contrast, the profiles of cells exposed to plastic for 60 and 180 min were most similar to each other as compared to all other samples.

A large number of genes (607) was regulated during the infection process as compared to the common reference (Table S4). Some of these (147) were also regulated on plastic, and therefore may be involved in adaptation to the common environmental conditions of the experiment (temperature, CO_2_ medium etc.) or may be associated with particular transcriptional programs, for example the yeast-to-hypha transition [5], [12]. However, we also monitored a specific transcriptional response to epithelial cells. The transcript levels of 547 *C. albicans* genes changed more than two-fold in cells co-incubated with epithelial cells at one or more time points compared to plastic (Table S5). Interestingly, most of these epithelial-specific responses were monitored at the early adaptation phase: the mRNA levels of 266 genes were significantly altered in response to epithelial cells at time point 20 min (compared to plastic), indicating that *C. albicans* rapidly senses and responds to epithelial cells (Table S5). The number of oral cell/plastic differentially regulated genes decreased during the experiment to 206 genes at 60 min and 186 at 180 min. Compared to plastic, a total of 24 genes were up- or down-regulated at all three time points, 20 genes at both 20 min and 60 min or 180 min, and 23 genes at both 60 min and 180 min (Fig. 4A). This indicates that a number of genes were specifically expressed due to contact with epithelial cells and that the profiles changed dynamically during the infection process.

Hierarchical clustering of all transcriptional profiles of *C. albicans* cells from all three time points either in contact to plastic or epithelial cells showed that profiles from *C. albicans* in contact to plastic at time points 60 and 180 min were most similar to each other, but different from the cells in contact to plastic at 20 min (Fig. 4B). In contrast, the expression profiles of *C. albicans* in contact to epithelial cells were not similar to each other at all three time points possibly indicating dynamic changes during the fungal-epithelial interactions. Furthermore, these profiles clustered distinct from the plastic control at all three time points, indicating a specific response of *C. albicans* to epithelial cells. In fact, we identified large sets of genes specifically responsive to epithelial cells. Some of these are discussed below.

### Expression of genes involved in protein synthesis, nutrient uptake and metabolism

The need for *C. albicans* to rapidly adapt to changing environmental conditions during the initiation of infection was reflected by the up-regulation of numerous genes involved in protein synthesis, including genes coding for ribosomal proteins (*RPS10, RPS14B, RPP2A, RPP2B, RPL38, 39,* and *82, MRPL8*), RNA helicases (*HAS1*) and transcriptional activators (*CTA26*) (Table S5). Interestingly, genes encoding key enzymes of the glyoxylate cycle and of gluconeogenesis (*ICL1*, *MLS1*, *PCK1*, *FBP1*) were strongly induced upon contact with epithelial cells in comparison to the YPD pre-culture (Table S4), but were not differentially expressed between oral cells and plastic. This suggests that, although *C. albicans* up-regulates alternative carbon assimilation pathways upon infection, this activity is not specifically induced by the presence of epithelial cells. We observed no indication of nitrogen starvation since genes coding for proteins involved in nitrogen transport such as amino acid permeases (*GAP1*) or oligopeptide transporter (*OPT8*) were down-regulated in *C. albicans* cells in contact with epithelial cells compared to plastic. Similarly, iron did not appear limiting as several genes known to be induced under high iron conditions such as *BIO2* and *PGA62* [16] were up-regulated compared to plastic. Concurrently, genes induced by low environmental iron, such as the transcription factor gene *MAC1*, the ferric reductase genes *FRE10* and *CFL2*, the high-affinity iron permease gene *FTR1*, the siderophore transporter genes *SIT1* [16] and *CYB2*, a gene transcriptional regulated by iron [16], were down-regulated in *C. albicans* cells in contact with epithelial cells compared to plastic. In contrast, the accessibility of other trace elements such as phosphate, copper and zinc seem to be limited during oral epithelial infection indicated by the up-regulation of a phosphate transporter (*PHO87*), the copper transporter gene *CRP1* and the zinc transporter gene *ZRT2*.

### Expression of stress response genes

Expression patterns indicated a stress response due to contact with epithelial cells or exposure to epithelial cellular content. For example, the genes *JIP5*, *PWP2* and *MPP10*, known to be down-regulated as part of the core stress response [17], [18], were down-regulated at 60 min. Similarly, *CDR4*, known to be up-regulated as part of the core stress response, was up-regulated at 60 min during contact with epithelial cells as compared to plastic. Together, this indicates that, at 60 min, *C. albicans* rapidly encounters an acute environmental insult; although, because of the non-specific responsive nature of these genes, their regulation does not indicate what stress that might be. It may be that *C. albicans* faces reactive oxygen species as *SOD5*, which play a protective role against oxidative stress, is up-regulated during contact with oral cells compared to plastic and strongly induced (up to 40 fold) compared to YPD. *YHB1*, which is involved in the detoxification of reactive nitrogen species was also induced, supporting the view that *C. albicans* faces nitrosative stress during interactions with epithelial cells [15]. However, *YHB1* is also transcriptionally up-regulated by oxidative stress [17], so a clear nitrosative stress response cannot be drawn from this data. Finally, *HSP70*, known to play a role in response to beta-defensins, was up-regulated in response to epithelial cells after 180 min of co-incubation with epithelial cells.

### Expression of genes associated with epithelial adhesion, invasion and damage

Many of the mutants which we had tested for epithelial infection (Fig. 1, Table S1) lacked genes encoding regulatory factors. Although such genes can play important roles under certain environmental conditions, differential expression levels may not be detected by microarray analysis due to transient expression and tight negative feedback. Indeed, despite the critical role of the cAMP-PKA-Efg1 pathway in mediating epithelial interactions, only the gene encoding Tec1 was significantly up-regulated during contact with oral epithelial cells as compared to YPD (Table S4). However, a number of target genes of Efg1 have been previously identified during hyphal growth and a set of 38 genes relies on the presence of *EFG1* [19]. We therefore analyzed the expression profile of these 38 genes during oral epithelial infection. Remarkably, 39% of these Efg1-target genes were up-regulated during infection. *IHD2*, orf19.1862, orf19.1654, *POL93* and *SAP6* were up-regulated compared to YPD; *ADH5*, orf19.1691 and orf19.3461 were up-regulated compared to plastic; *ECE1*, *HWP1*, *IHD1*, *IRO1*, orf19.3384, *PLB5* and *SOD5* were up-regulated compared to both YPD and plastic.

Similarly, *RIM101*, which encodes a pH-responsive transcriptional regulator, was required for all stages of epithelial interactions (Fig. 1) but not transcriptionally regulated during infection. However, numerous Rim101 target genes, such as *ZRT1* (encoding a zinc transporter), *PGA10*/*RBT51* (involved in haemoglobin utilization), *PHR1* and *PRA1* (encoding surface antigens) were more highly expressed by *C. albicans* cells in contact with epithelial cells than plastic.

We had also demonstrated the importance of the hyphal-associated cell surface adhesins (*ALS3* and *HWP1*) for oral infection. Reassuringly, both of these genes, along with numerous other hyphal-associated genes were more highly expressed during contact with epithelial cells than YPD (Table S4). Interestingly, many hyphal-associated genes, including *ALS3*, *HWP1*, *ECE1*, *SOD5*, *PHR1*, *PRA1*, and *RBT1* were defined as transcriptionally up-regulated in *C. albicans* cells in contact with epithelial cells compared to contact to plastic (Table S5), despite the fact that under both of these conditions *C. albicans* grew as hyphae. These data indicate that hyphal-associated genes can be expressed at different levels by hyphae growing on different surfaces. Moreover, *ECM33*, which also encodes a cell surface adhesin, but which has not been previously described as hypha-associated, was up-regulated during contact with epithelial cells compared to plastic, but not compared to YPD.

Factors involved in glycerol homeostasis (*GPD2*, *GPP1*, *PMT2*), morphogenetic plasticity (*VPS11, GPP1*, *EED1*), detection of physical contact (*MKC1*), or factors which are important for maintenance of cell polarity, directed growth and thigmotropism (*RSR1, BUD2*) were also important for epithelial infection (Fig. 1). However, most of these genes did not exhibit significant regulation. Due to the important role of these genes in damage of epithelium (which was measured at 24 h), it is possible that they are not up-regulated until later time-points, outside the 3 h of our transcriptional analysis.

### Functional characterization of epithelial-induced genes

**Figure 5:**
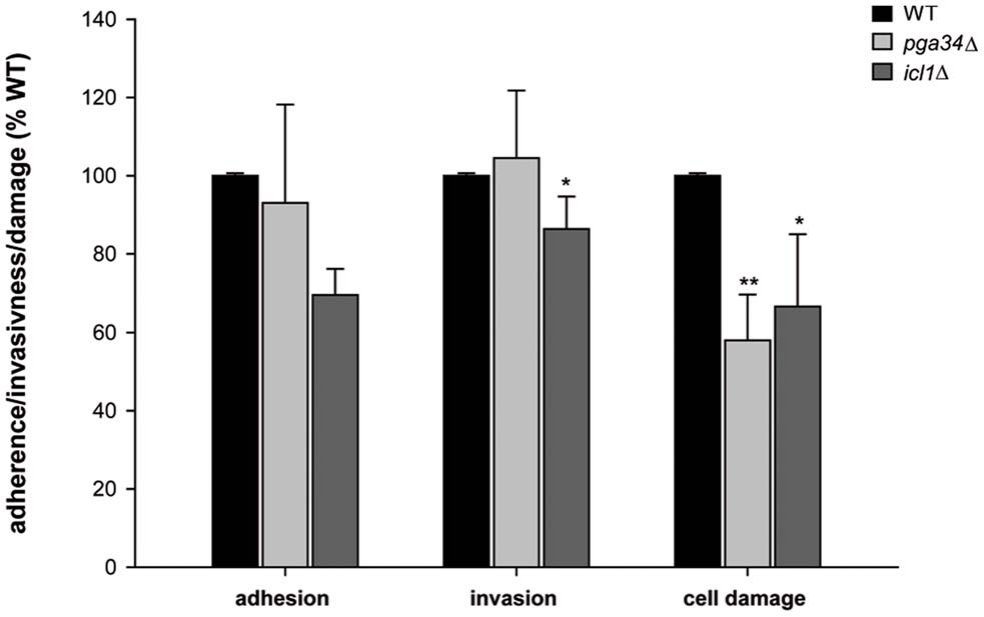
Adhesion, invasion and damage properties of selected mutants lacking epithelial infection up-regulated genes. *C. albicans* mutant strains lacking *ICL1* and *PGA34* showed wild type levels of adhesion and/or invasion. However, *icl1*Δ showed significantly reduced invasion and damage and *pga34*Δ showed significantly reduced damage. The values are calculated as percentage of adherent/invasive cells or damage compared to the corresponding wild type (100%). **, significant difference compared to the corresponding wild type (WT) (*p<*0.01).

Our transcriptional profiling of *C. albicans* oral epithelial infection had confirmed the expression of some of the genes which we had shown to be involved in host-pathogen interactions (above); however, we also observed up-regulation of genes involved in alternative carbon assimilation, stress response and a large number of genes of unknown function (above). We therefore selected eight representative genes for further functional analysis during oral epithelial infection. These were: *ICL1* (encoding an isocitrate lyase of the glyoxylate cycle, required for alternative carbon metabolism), *YHB1* (encoding a nitric oxide dioxygenase required for nitric oxide detoxification), *SOD5* (encoding a superoxide dismutase, required for oxidative stress tolerance), *PGA34*, orf19.851, orf19.3459, orf19.3600 and orf19.6837 (all of which encode proteins of unknown function). *sod5*Δ, *icl1*Δ and *yhb1*Δ mutants had already been constructed [20], [21], [22], [23]. For the other five genes we constructed homozygous mutants as described in *Material and Methods*. Deletion of *SOD5*, *YHB1*, orf19.851, orf19.3459, orf19.3600 and orf19.6837 did not alter epithelial damage (data not shown). However, *pga34*Δ and *icl1*Δ mutants exhibited reduced epithelial damage (Fig. 5). *pga34*Δ adhered to and invaded epithelial cells at wild type levels, whilst *icl1*Δ also exhibited reduced invasion.

These data suggest that a functional glyoxylate cycle is essential for epithelial invasion and damage. Moreover, it would appear that genes of unknown function can play important roles during oral infection.

## Discussion

### Cellular dissection of the early stages of *C. albicans*-epithelial interaction

Microbial invasion of non-professional phagocytic host cells can occur via two general mechanisms: active penetration or induced endocytosis. Plant pathogenic fungi can actively penetrate plant cell walls by producing specialized structures (called appressoria), hydrolases and high turgor pressures [24], [25]. Parasites, such as members of the apicomplexa group, can actively penetrate into host cells using actin-mediated forces [26]. Bacteria, on the other hand, cannot actively penetrate host cells, but have developed several strategies to cause up-take by induced endocytosis [26], [27]. Therefore, different pathogenic microbes have developed different mechanisms for invading host cells.

*C. albicans* is capable of invading epithelial cells via both induced endocytosis and active penetration [10], [15], [28].

### Molecular dissection of oral epithelial infection

The only defined molecular mechanism of epithelial invasion by *C. albicans* is Als3-E-cadherin mediated induced endocytosis. We therefore systematically assessed the role of 26 genes (Table S1) in adherence to, invasion and damage of oral epithelial cells. These genes were carefully selected based on their described or predicted function, which we hypothesized to be important for the different stages of epithelial infection. Although some of these mutants had previously been tested for certain stages of epithelial interaction, we here provide an infection-orientated systematic functional analysis, supported by global transcriptional profiling of *C. albicans* infection of epithelial cells. Moreover, we selected additional genes which we identified as transcriptionally up-regulated in *C. albicans* cells in contact with epithelial cells and assessed the effect of deletion of these genes on epithelial invasion and damage. Table S3 summarizes the epithelial adhesion, invasion and damage phenotypes of all mutants as described previously and as demonstrated in the current study. In the following section we describe some of the trends that we observed based on our functional analysis and transcriptional profiling.

### Correlation with previous studies

Of the total of 34 mutants tested here, 13 had previously been characterized for epithelial interactions and overall our findings are in agreement with previous studies (Table S3). With the exception of *tpk1*Δ, the only observed discrepancies were at the invasion stage: we observed normal invasion by *bud2*Δ, *rsr1*Δ and *cka2*Δ. Brand *et al*. (2008) reported reduced invasion potential of *bud2*Δ and *rsr1*Δ into TERT-2 epithelial cells following 8 h incubation [29]. Chiang *et al*. (2007) reported reduced invasion of *cka2*Δ into FaDu oral epithelial cells following 90 min incubation [30]. Conversely, we observed reduced epithelial adhesion, invasion and damage for both *tpk1*Δ and *tpk2*Δ strains, whereas Park *et al*. (2005) reported no difference between wild type and *tpk1*Δ for adhesion to (90 min), invasion (90 min) or damage (3 h) of FaDu epithelial cells and reduced adhesion and damage for *tpk2*Δ [31]. We conclude that these observed invasion differences are due to the different oral epithelial cell line (TR-146) and incubation time (3 h) used in the present study.

A transcriptional profiling study of *C. albicans* strains CAI4-URA3 and a clinical isolate (7392) infecting FaDu oral epithelial cells has recently been described by Park *et al*. (2005) [32]. In this study, the authors identified 51 genes as transcriptionally up-regulated during contact with oral epithelial cells compared to plastic. Despite the fact that different *C. albicans* strains, cell culture medium, epithelial cell lines and time points were analyzed by Park *et al*. (2005) [32], we also observed the induction of four genes in *C. albicans* cells in contact with epithelial cells that were in common with the previous study. These genes, shown to be expressed in both studies were: *MET1*, *SEC14*, orf19.631 and orf19.6931. Interestingly, *SEC14*, which encodes an essential phospholipid transfer protein, involved in membrane trafficking and the production of secretory vesicles from the Golgi apparatus [33] was also reported as transcriptionally up-regulated during infection of oral RHOE tissue [15] (Table S5), suggesting a core role for secretory vesicle transport during oral infection. Indeed many of the genes which we observed as up-regulated during epithelial monolayer infection, including *ALS3*, *HWP1*, *ICL1*, *PHR1*, *SOD5*, and *YHB1* were also shown to up-regulated in samples from patients suffering from oral candidosis [15], suggesting that our epithelial monolayer model does reflect certain aspects of oral infection.

Interestingly, four of these genes were also shown to be required for interactions with epithelial cells: *als3*Δ, *hwp1*Δ, *icl1*Δ (this study) and *phr1*Δ [34] were attenuated during invasion and/or damage of human epithelia, further indicating that *in vitro* analysis can mirror the situation *in vivo*.

### The core elements of the cAMP-PKA-Efg1 pathway are required for all stages of oral infection

The expression of cell surface adhesion factors, as well as the yeast-to-hyphal transition itself, are governed by a network of signal transduction pathways. Interestingly, mutants lacking each of the components of the cAMP-PKA pathway which we investigated (Ras1, Tpk1, Tpk2, Efg1), as well as Rim101 and Hgc1 [34], which also signal through Efg1, together with the Efg1 targets, *TEC1* and *CZF1*, displayed reduced adhesion, invasion and damage of oral epithelial cells (group 2). The components of this pathway are required for both hyphal morphogenesis [35] and the correct expression of cell surface adhesion factors including Als3 and Hwp1. The attenuated adhesion, invasion and damage of these mutants is therefore likely due to combined defects in morphogenesis and the expression of other factors.

Moreover, of the 38 genes which have been reported to rely on the presence of Efg1 for expression during hyphal growth [19], 39% were transcriptionally up-regulated in our model. Combined with the phenotypes of the *efg1*Δ mutant and mutants of up-stream regulators of Efg1, these data suggest that the output of the cAMP-PKA pathway is important for all stages of interactions with oral epithelial cells. Therefore, this underscores the importance of hyphal morphogenesis, together with the associated transcriptional programs for this type of infection.

Although Ras1 is also an upstream element of the Cek1 MAPK pathway, which regulates morphology and hyphal associated genes under certain conditions, signaling through this pathway does not appear to be as important for invasive growth under the conditions tested here. This is evident since a *cph1*Δ mutant, which lacks the terminal transcription factor of the Cek1 MAPK pathway, retained wild type levels of adhesion, invasion and damage. This provides further evidence that defects observed upon *RAS1* deletion were mainly due to disruption of the cAMP-PKA pathway.

Surprisingly, a mutant lacking Cph2 was not significantly attenuated in adhesion, invasion or damage. Cph2 is known to regulate Tec1 [36], which in turn controls Bcr1 and thus Als3 and Hwp1 expression [37]. However, since hyphal formation of the *cph2*Δ mutant was also not altered in our model (Table S4), we concluded that *cph2*Δ hyphae likely express Als3, Hwp1 or other adhesion factors similar to the wild type under the conditions investigated.

### Fungal cell surface adhesins mediate initial attachment, but play differential roles in invasion

In this study we analyzed the behavior of mutants lacking two major adhesion factors (Als3 and Hwp1), as well as their direct upstream activator (Bcr1) during interactions with epithelial cells. Our microarray analysis demonstrated that both *ALS3* and *HWP1* were transcriptionally up-regulated during contact with oral epithelial cells and that mutants lacking either *HWP1* or *ALS3* have reduced potential to adhere to oral cells as compared to wild type, confirming previous data from other studies that Als3 and Hwp1 are major adhesins of *C. albicans* [8], [38]. Deletion of *BCR1* resulted in an even stronger adhesion defect, in line with the role of Bcr1 as a direct transcriptional activator of adhesin-encoding genes (including both *ALS3* and *HWP1* – [37]). Interestingly, while deletion of the multi-functional (adhesin, invasin and ferritin receptor) *ALS3* gene inhibited adhesion and invasion as well as subsequent damage (group 2), *bcr1*Δ and *hwp1*Δ mutants exhibited reduced adhesion but invaded at rates similar to the wild type (group 5). These data fit with the idea of Als3 as both an adhesin and invasin. Furthermore, it would appear that, at the invasion phase, either Bcr1-dependent expression of *ALS3* is bypassed or that compensatory induction of other invasion factors occurs. Indeed, it has been shown that *als3*Δ cells can form biofilms *in vivo*, but not *in vitro* [39], demonstrating that additional host-associated stimuli can bypass dependency on Als3 for biofilm formation. Although capable of invading epithelial cells, *bcr1*Δ and *hwp1*Δ caused reduced damage compared to the wild type. This may be linked to delayed adhesion, resulting in perturbed epithelial interactions at later stages. However, it is also possible that these two factors play additional roles in epithelial destruction at later time points. For example, Bcr1 may be required for the expression of other cell surface components which contribute to epithelial damage.

### The physical contact sensing/response machinery and morphogenic plasticity play specific roles during interactions with epithelial cells

*PMT2* and *ICL1* were the only genes specifically required for invasion and damage. Deletion of a single copy of *PMT2*, which encodes a protein mannosyltransferase, results in numerous physiological defects including reduced growth, protein mannosylation (possibly including that of cell surface adhesins), cell wall β-1,6-glucan and mannoprotein levels, glycerol content and a significant down-regulation of *GPP1* and *GPD2*, and defective expression of secreted proteases [14], [40], [41]. Therefore, a combination of these defects may account for the observed reduction in epithelial invasion and damage. Icl1, on the other hand, specifically mediates the conversion of isocitrate to succinate and glyoxylate. It is unclear whether *C. albicans* genuinely relies on alternative carbon sources for growth during our *in vitro* model of oral infection, as is the case during systemic infection [23], or if the glyoxylate cycle is required for the biosynthesis of metabolic intermediates involved in invasion, analogous to glycerol-mediated turgor pressure of plant pathogens [25].

Group 4 and 5 damage-associated mutants exhibited specific defects in epithelial interactions: mutants lacking *MKC1*, *GPD2*, *GPP1*, *EED1*, *CKA2*, *BUD2*, *RSR1*, and *PGA34* invaded epithelial cells at similar rates to the wild type but were defective in oral epithelial damage. Moreover, *gpd2*Δ, *gpp1*Δ, *cka2*Δ, *bcr1*Δ, *hwp1*Δ, *bud2*Δ and *rsr1*Δ all exhibited significantly reduced invasion into Caco-2 enterocytes, but not into TR146 oral epithelial cells. Furthermore, *bud2*Δ, *cka2*Δ, *gpd2*Δ, *gpp1*Δ and *mkc1*Δ were attenuated for damage of oral epithelial monolayers but not multilayers.

*CKA2* encodes a conserved catalytic subunit of the CK2 protein kinase which has been implicated in the calcineurin pathway in *C. albicans* [42]. Because of its central role in governing calcium homeostasis, the calcineurin pathway likely plays an important role in governing hyphal orientation [43]. Similarly, *MKC1* encodes a mitogen-activated protein kinase of the cell wall integrity pathway and is involved in contact sensing-mediated invasive growth [44]. The Ras-GTPase, Rsr1 and its cognate GTPase activating protein, Bud2 have both been shown to be directly involved in hyphal orientation and thigmotropism [29]. *GPP1* and *GPD2* both encode enzymes of glycerol biosynthesis and glycerol accumulation is essential for generating turgor pressure in fungi [25]. Although we were only able to directly measure reduced glycerol content of the *gpp1*Δ mutant under osmotic stress conditions, it is likely that Gpd2 also contributes to glycerol biosynthesis, possibly only under specific conditions; alternatively, Gpd2 may promote glycerol accumulation specifically at the hyphal tip.

*EED1* is dispensable for initial germ tube formation but essential for the maintenance of hyphal elongation [15]. Finally, *PGA34* encode a secreted and cell surface-associated protein of unknown function.

Taken together, these data provide a portrait of the processes involved in epithelial damage. Firstly, the specific epithelial damage defects of these group 4 and 5 mutants demonstrate that initial epithelial invasion is not sufficient to cause tissue destruction, but rather suggests a model whereby, following adhesion and initial internalization, *C. albicans* relies on a combination of active penetration, directed hyphal extension, glycerol homeostasis and the expression of novel pathogenicity factors for deeper invasion and for further inter-epithelial invasion and dissemination. Secondly, the fact that mutants defective in thigmotropism and glycerol homeostasis (group 4 and 5) were able to invade oral epithelial cells (induced endocytosis and active penetration), but unable to invade enterocytes (active penetration only) suggests that a combination of turgor pressure (glycerol accumulation) and directed hyphal growth is specifically required for fungal penetration at distinct stages of infection.

In summary, our systematic molecular analysis provides a comprehensive picture of the processes governing epithelial infection: from the fundamental involvement of the core signaling pathways and hyphal formation to all stages of epithelial interactions, to the specific roles of morphogenic plasticity in mediating epithelial destruction.

## Materials and Methods

### Strains and media

*Candida albicans* strains SC5314 [20], [45], BWP17 [46] carrying CIp30 [47], RM1000 [48] and CAI-4 carrying CIp10 [49] were used as wild type controls. The genotypes of all *C. albicans* strains used in this study are listed in Table S6. All strains were maintained on YPD plates (1% peptone, 1% yeast extract, 2% glucose, 2% agar). For use in the experiments, *C. albicans* cells from an overnight YPD culture were either diluted to OD_600_ = 0.2 in fresh liquid YPD medium and grown to log phase for a further 4 h at 30°C or semi-synchronized for further 24 h in liquid YPD medium at 30°C [50]. *C. albicans* cells were then harvested by centrifugation, counted with a hemacytometer and adjusted to the desired concentration in serum-free DMEM medium (containing 2 mM L-glutamine) immediately prior to the experiment.

### Mutant strain construction

Strain BWP17 [46] was used to create mutant strains. Null mutants lacking orf19.691 (*GPD2*), orf19.5437 (*GPP1*), orf19.851, orf19.2833 (PGA34), orf19.3459, orf19.3600, orf19.6837 were constructed as previously described using pFA-His and pFA-Arg plasmids [51]. Briefly, cassettes for transformation were amplified with primers which included >100 bp of gene specific sequences at their 5′ end and the corresponding annealing regions to the pFA vector at the 3′ end. Disruption primers are listed in Table S7. Finally, *URA3* was integrated into the *RPS1* locus via integration of CIp10 [52].

Transformation was performed according to Walther and Wendland [53]. A parental strain was created by integrating CIp30 (*URA3*; *HIS1*; *ARG4*) [47] into the *RPS1* locus of BWP17. CIp10 and CIp30 were kindly provided by Alistair Brown, Aberdeen. All experimental strains were compared with the wild type (SC5314) and parental strain (BWP17+ pCIp30). All gene manipulations and mutants produced in this study were confirmed by PCR and Southern blot analysis.

### Epithelial cells

The colon adenocarcinoma derived cell line Caco-2 was obtained from the American Type Culture Collection (ATCC) (HTB 27). Caco-2 cell monolayers displayed several morphological and functional characteristics of mature enterocytes [54]. These cells were routinely cultured (passages 4 to 25) in DMEM medium supplemented with 10% FCS, 1 mM pyruvic acid, 2 mM L-glutamine and 0.1 mM non-essential amino acids (all media from Biochrom AG, Berlin, Germany), without antibiotics or antifungal agents. The squamous carcinoma of buccal mucosa derived epithelial cell line TR146 was obtained from Cancer Research Technology, London [55]. TR146 cells were routinely grown (passages 4 to 20) in DMEM medium with 10% FCS, 1 mM pyruvic acid and 2 mM L-glutamine, without antibiotics or antifungal agents. The RHOE for epithelial multilayers was based on cultured TR146 cells and supplied by SkinEthic Laboratories (Nice, France). The RHOE was maintained in serum-free Maintenance medium (SkinEthic), on a 0.5 cm^2^ microporous polycarbonate filter (insert) [56]. All cell types were maintained in a humidified incubator at 37°C in 5% CO_2_. For standard experiments, 1×10^5^ of TR146 or Caco-2 cells, respectively, were seeded onto acetic acid treated 15 mm diameter glass coverslips previously placed in 24-well plates and cultured up to 21 days post-seeding.

### Adherence assay

*C. albicans* adherence to oral epithelial cells was determined using fluorescence microscopy. TR146 cells were grown on 15 mm glass coverslips for two days and inoculated with exactly 1×10^5^ *C. albicans* cells (without centrifugation). Next the cells were co-incubated for 1 h in DMEM medium without FBS. For the time course of *C. albicans* adherence to oral epithelial cells, *C. albicans* cells were incubated on a TR146 monolayer for 20 min, 1 h, 2 h or 3 h at 37°C and 5% CO_2_. After co-incubation, non-adherent cells were removed by extensively rinsing five times with PBS and fixed with 4% paraformaldehyde. Next, host cells were permeabilized with 0.5% Triton X-100. Adherent *C. albicans* cells were stained with calcofluor white and quantified by epifluorescence (Leica DM5500B, Leica DFC360 FX) using a filter set to detect calcofluor white. The number of adherent cells was determined by counting of at least 100 high power fields of 320 µm ×240 µm size. Exact total cell numbers were calculated based on the quantified areas and the total size of the cover slip. Each condition was tested in duplicates, and at least three separate experiments were performed.

### Invasion assay

The number of *C. albicans* cells that invaded epithelial cells was determined using a protocol derived from Park et *al*. (2005) [31]. Briefly, epithelial cells were grown on 15 mm diameter glass coverslips for 2 days (monolayers of TR-146 cells) or 15–21 days post-seeding (monolayers of differentiated Caco-2 cells). The monolayers were infected with 1×10^5^ log phase yeast cells of *C. albicans* and placed in a humidified incubator. After 3 h incubation, the medium covering the cells was aspirated and monolayers were rinsed three times with PBS to remove fungal cells, which were not associated with epithelial cells. Note that by 3 h incubation virtually all (>98%) wild type fungal cells become attached to the monolayer. For epithelial membrane staining, the cells were incubated with Vybrant DiI cell-labeling solution (Molecular Probes, USA) 1∶20 in DMEM for 5 min in a humidified incubator at 37°C. Next, the epithelial cells were fixed with 4% paraformaldehyde (Roth). All fungal cells remaining adherent to the surface of the epithelial cells were stained for 1 h with green-fluorescent Alexa Fluor 488 conjugate of succinylated concanavalin A (Con A) (Invitrogen) (note: ConA stains only the extracellular, non-invaded fungal elements). After rinsing with PBS, epithelial cells were permeabilized in 0.5% Triton X-100 in PBS for 5–10 min. Next, complete fungal cells (*i.e.* invaded and non-invaded) were stained with calcofluor white. The coverslips were then rinsed with water, mounted inverted onto slides, and the stained cells were visualized with epifluorescence (Leica DM5500B, Leica DFC360 FX) using filter sets to detect Alexa Fluor 488, 568 and calcofluor. The percentage of invading *C. albicans* cells was determined by dividing the number of [partially] internalized cells by the total number of adherent cells. At least 100 fungal cells were counted on each coverslip and all experiments were performed in duplicates on at least three separate occasions. Images were taken with a Leica Digital Camera DFC360 FX.

### Damage assay

Epithelial cell damage caused by different *C. albicans* strains during interaction with TR146 cells was determined by the release of lactate dehydrogenase (LDH) into the surrounding medium following 24 h uninterrupted co-incubation with *C. albicans*. TR146 monolayers were grown to 95% confluency in 96 well culture plates and infected with 2×10^4^ cells in DMEM with 1% FCS and placed in a humidified incubator. For control samples, TR146 cells were incubated with DMEM medium only or DMEM containing 0.5% Trion X-100; additionally, *C. albicans* cells were seeded without epithelial cells. For measuring the damage of epithelial multilayers, the RHOE was infected with 2×10^6^ *Candida* cells in 50 µl PBS. Non-infected controls contained 50 µl PBS alone. After 24 h extracellular LDH release into the medium was measured spectrophotometrically at 492 nm using the Cytotoxicity Detection Kit (LDH) from Roche Applied Science according to the manufacturer's instructions. The percentage cytotoxicity of epithelial cells infected with *C. albicans* cells was calculated as follows: experimental LDH release minus background cells minus background *Candida*/mean maximal LDH release minus background cells and compared to 100% WT. Compared to experimental LDH release by Triton X-100 treatment, the tested wild type strains released 41% LDH. All experiments were performed in triplicates for each condition and repeated three times. For statistical analysis, *p*-values <0.05 were considered as significant.

### Isolation of *C. albicans* RNA

*C. albicans* cells suspended in pre-warmed DMEM medium were added to 6 well polystyrene tissue culture plates containing oral epithelial cells. As a control, organisms were added to empty tissue culture plates that did not contain host cells (hereafter called plastic). In all experiments, the final concentration of organisms was 1×10^6^ per well and the same RNA extraction procedure was used for both the experimental and control conditions. Fungal cells were incubated with host cells or plastic for 20, 60 or 180 min. At the end of each incubation period, the medium containing non-adherent fungal cells was removed. To reduce the amount of host cell RNA and to stop RNA transcription, PeqGold RNApure reagent (Peqlab) was added to each well and afterwards snap frozen in liquid nitrogen. The total time from rinsing cells to freezing in liquid nitrogen was less than 5 min. After thawing, fungal cells were collected by centrifugation and supernatants were removed to decrease the amount of human RNA. Next, the pellet was resuspended in AE-buffer (50 mM Na-acetate pH 5.3, 10 mM EDTA, 1% SDS). After addition of phenol/chloroform/isoamylalcohol (25∶24∶1) cells were heated for 5 min 65°C and immediately shock frozen. Next, cells were thawed on ice and fungal RNA was precipitated by addition of isopropanol and sodium acetate and incubation at −20°C over night. The quality of RNA was determined using a Bioanalyzer (Agilent Inc.) and the quantity was measured with nanodrop ND1000 (Peqlab).

### cDNA labeling and microarray hybridization

Preparation and labeling of cDNA and hybridization of microarrays were performed following standard protocols. Total RNA was linearly amplified and labeled using the ‘Low RNA Input Fluorescent Linear amplification Kit’ (Agilent Technologies, Santa Clara, CA, USA). Briefly, 10 µg of total RNA was reverse transcribed with Superscript II reverse transcriptase (Invitrogen) in the presence of 5-(3-aminoallyl)-2′-deoxyuridine 5′- triphosphate (aa-dUTP), using both oligo dT and random primers (Stratagene). For transcriptional profiling, we used *C. albicans* microarrays (Eurogentec, Seraing, Belgium) as described [21]. Sample RNA, Cy5- labeled) was co-hybridized at a ratio of 1∶1 with a ‘common reference’ (RNA from SC5314 grown in YPD, mid-log phase, 37°C, Cy3-labelled), making dye swap controls unnecessary. Slides were hybridized, washed and scanned as described [21]. The arrays were visualized with 428TM array scanner (Affymetrix). At least three hybridizations were performed for each time point. Data normalization (LOWESS) and analysis were performed using GeneSpring GX 10.0.1 software (Agilent Technologies). Reliable expression of genes was defined as normalized expression of present genes that did not vary more than 1.5 standard deviations within replicate arrays. Genes with a signal value greater than the cut-off 50 were stated as specifically regulated and false positive were excluded according to the Benjamini-Hochberg Procedure [57]. Entities where at least 50% of the samples in any 3 out of 6 conditions have values within the cut-off. Genes which showed 1.5- to 2-fold changes compared with the plastic control or the common reference were considered as ‘increased’ and ≥2-fold as ‘up-regulated’. All microarray data are MIAME compliant and raw data have been deposited at ArrayExpress (Accession number: E-MEXP-3015).

### Real-time RT-PCR

The validity of the microarray results was assessed for 8 selected key genes by real-time RT-PCR using SYBR green detection in a Mx3000P QPCR System (Agilent technologies). RNA samples extracted for microarray analysis were used. First genomic DNA was digested with Baseline-ZERO™ DNase (Epicentre Biotechnologies). Complete removal was checked by PCR for each RNA sample. First-Strand cDNA were synthesized with the SuperScript® III First-Strand Synthesis Kit (Invitrogen) following the manufacturer's protocol. Genes investigated were *CRP1*, *OPT9*, orf19.6835, *PGA37*/orf19.3923, *GPP1*, orf19.3600, *ECE1* and *ALS3*. The primers used in these experiments are listed on Table S7. Each primer pair was tested for cross reactivity with epithelial cDNA. Relative transcript abundance was determined with the 2 –ΔΔCt method [58] using the transcript level of Ca*ACT1* and Ca*EFB1* as internal controls. For the time points 60 min and 180 min, 8 genes were analyzed in at least three biological replicates and the results were combined. Overall, the change in expression of all genes tested by qRT-PCR was in agreement with the direction of fold change as determined by microarray analysis.

### Measurement of intracellular glycerol

The intracellular glycerol content of *C. albicans* SC5314, *gpd2*Δ and *gpp1*Δ were measured with a commercial quantitative colorimetric determination kit (EnzyChrom™Glycerol Assay Kit, BioAssay Systems, Hayward, USA) following the manufacturer instructions. Briefly, cells were grown overnight in selection media (SD). The overnight culture was re-inoculated in YPD to OD_600_ = 0.2 and grown for additional 4 h at 30°C. Next, cells were treated with 0.5 M NaCl for 45 min by adding 10 ml of 1 M NaCl in YPD, or treated with YPD only as control. Afterwards, cells were collected by centrifugation and washed twice with water. One ml was heated at 95°C for 10 min and then centrifuged at 500 rpm for 30 s. The supernatant was used for glycerol determination. Glycerol concentrations were normalized to the wet weight of each pellet.

### Statistical analyses

The data were analyzed using a Student's T-test to compare means. For these analyzes, *p* values of <0.05 were considered to be significant. For some experiments we chose to set the level of significance for tests at *p<*0.01.

## Supporting Information

Table S1 Description of genes selected for analyses in this study.
(DOC)

Table S2 Morphology of *C. albicans* wild type and mutant strains (hyphal, pseudohyphal and yeast cells formation in %) and length of the formed filaments following 3 h incubation on epithelial monolayers.
(DOC)

Table S3 Summarized phenotypes of *C. albicans* wild type and mutant strains during interaction with epithelial cells. Comparison of published data to adhesion, invasion and damage properties of all strains investigated in this study.
(DOC)

Table S4 *C. albicans* genes significantly up- and down-regulated in response to oral epithelial cells as compared to YPD cultured *C. albicans* cells (common reference) at 20, 60, and 180 min.
(XLS)

Table S5 *C. albicans* genes with significant changes in transcript levels in response to oral epithelial cells as compared to plastic by microarray analyses at different time points.
(XLS)

Table S6 List of the genotypes of *C. albicans* strains used in this study.
(DOC)

Table S7 Primers used in this study for gene disruption and quantitative real-time RT-PCR.
(XLS)
