# Supplementary material for: From Attachment to Damage: Defined Genes of Candida albicans Mediate Adhesion, Invasion and Damage during Interaction with Oral Epithelial Cells
Source: PLoS One. 2011 Feb 23;6(2):e17046. doi: 10.1371/journal.pone.0017046 (PMC3044159; doi:10.1371/journal.pone.0017046)
Supplement: Table S1 — Description of genes selected for analyses in this study. (DOC) [file pone.0017046.s001.doc]

**Table S1. Description of genes selected for analyses in this study.**

| **Gene** | **Description** | **Mutant phenotype for hyphal growth, biofilm formation and *in/ex vivo* virulence** | **Reference** |
| --- | --- | --- | --- |
| *ALS3* | Adhesin, invasin, ferritin receptor | normal filamentous growth, abnormal biofilm formation, decreased virulence | [1,2,3,4] |
| *BCR1* | C2H2 zinc finger transcription factor, biofilm and cell wall regulator | normal filamentous growth, abnormal biofilm formation, normal virulence | [5,6] |
| *BUD2* | GTPase activating protein for Rsr1, involved in polar bud site selection and hyphal growth guidance, thigmotropism | abnormal filamentous growth | [7] |
| *CKA2* | Catalytic subunit (alpha-subunit) of protein kinase CK2, central role in governing calcium homeostasis | normal filamentous growth, decreased virulence | [8,9] |
| *CPH1* | Transcription factor required for mating and hyphal growth on solid media | normal filamentous growth, abnormal biofilm formation, decreased virulence | [10,11,12,13,14] |
| *CPH2* | Myc family bHLH transcriptional activator of hyphal growth | normal filamentous growth, normal virulence | [15,16,17] |
| *CZF1* | Zink finger protein, regulator of hyphal growth | abnormal filamentous growth, normal virulence | [15,18] |
| *ECM33* | GPI-anchored cell wall protein, involved in cell wall integrity | abnormal filamentous growth, decreased virulence | [19] |
| *EFG1* | Transcription factor with bHLH required for hyphal growth and metabolism; cell-wall gene regulator | abnormal filamentous growth, abnormal biofilm formation, decreased virulence | [10,11,20,21] |
| *GPD2* | Similar to glycerol 3-P dehydrogenases | normal filamentous growth | [22] |
| *GPP1* | Putative glycerol 3-phosphatase | normal filamentous growth | [22] |
| *HGC1* | Hypha-specific G1 cyclin-related protein, regulation of hyphal morphogenesis | abnormal filamentous growth, decreased virulence | [23,24] |
| *HWP1* | Hyphal cell wall protein, adhesin | normal filamentous growth, decreased biofilm formation, decreased virulence | [5,25,26] |
| *HYR1* | GPI-anchored cell wall protein, hyphal-induced, macrophage-induced | normal filamentous growth | [27] |
| *IPF946 (EED1)* | Epithelial escape and dissemination | abnormal filamentous growth | [28] |
| *ICL1* | Isocitrate lyase of the glyoxylate cycle, involved in utilization of alternative carbon metabolism | normal filamentous growth, decreased virulence | [29] |
| *MKC1* | MAP kinase of the cell wall integrity pathway, contact induced filamentation | normal filamentous growth, abnormal biofilm formation, decreased virulence | [30,31,32] |
| *PLB1* | Phospholipase B | normal filamentous growth, decreased virulence | [33] |
| *PMT2* | Mannosyltransferase, involved in cell wall regeneration (β-1,6-glucan and mannoprotein levels) | homozygous mutant not viable, heterozygote abnormal filamentous growth, decreased biofilm formation | [34,35] |
| *RAS1* | RAS signal transduction GTPase; regulates cAMP and MAP kinase pathways | abnormal filamentous growth, decreased virulence | [36,37] |
| *RIM101* | Transcription factor involved in alkaline pH response | abnormal filamentous growth, decreased invasive growth, decreased virulence | [38,39,40] |
| *RSR1* | GTP/GDP cycling, involved in polar bud site selection and hyphal growth guidance, thigmotropism | abnormal polar bud site selection and hyphal growth guidance, thigmotropism, decreased virulence | [7] |
| *SOD5* | Copper- and zinc-containing superoxide dismutase, required for oxidative stress tolerance | normal filamentous growth, decreased virulence | [41] |
| *TEC1* | TEA/ATTS transcription factor involved in regulation of hypha-specific genes, regulates Bcr1 | abnormal filamentous growth, decreased biofilm formation, decreased virulence | [6,16,42,43] |
| *TPK1* | Catalytic subunit of cAMP-dependent protein kinase (PKA), isoform of Tpk2p | abnormal filamentous growth, normal virulence | [20,44,45] |
| *TPK2* | Catalytic subunit of the cAMP-dependent protein kinase A (PKA) | abnormal filamentous growth, reduced virulence | [20,45,46] |
| *TUP1* | Repressor of filamtous growth | hyperfilamentous growth, decreased virulence | [11,47,48,49] |
| *VPS11* | Involved in protein trafficking; putative role in vesicle-target membrane fusion, required for vacuole formation | abnormal filamentous growth, decreased virulence | [50,51] |
| *YHB1* | Nitric oxide dioxygenase, required in nitric oxide scavenging/detoxification | abnormal filamentous growth, decreased virulence | [52] |
| *orf19.851* | Protein of unknown function; transcription is negatively regulated by Rim101p (Eukaryot Cell 2(4):718-28) | / |  |
| *orf19.2833* | PGA34, putative GPI-anchored protein of unknown function; transcription is repressed in response to alpha pheromone in SpiderM medium | / |  |
| *orf19.3459* | Not characterized | / |  |
| *orf19.3600* | Not characterized | / |  |
| *orf19.6837* | Not characterized | / |  |

1. Almeida RS, Brunke S, Albrecht A, Thewes S, Laue M, et al. (2008) the hyphal-associated adhesin and invasin Als3 of Candida albicans mediates iron acquisition from host ferritin. PLoS Pathog 4: e1000217.

2. Hoyer LL, Payne TL, Bell M, Myers AM, Scherer S (1998) Candida albicans ALS3 and insights into the nature of the ALS gene family. Curr Genet 33: 451-459.

3. Phan QT, Myers CL, Fu Y, Sheppard DC, Yeaman MR, et al. (2007) Als3 is a Candida albicans invasin that binds to cadherins and induces endocytosis by host cells. PLoS Biol 5: e64.

4. Zhao X, Oh SH, Cheng G, Green CB, Nuessen JA, et al. (2004) ALS3 and ALS8 represent a single locus that encodes a Candida albicans adhesin; functional comparisons between Als3p and Als1p. Microbiology 150: 2415-2428.

5. Nobile CJ, Andes DR, Nett JE, Smith FJ, Yue F, et al. (2006) Critical role of Bcr1-dependent adhesins in C. albicans biofilm formation in vitro and in vivo. PLoS Pathog 2: e63.

6. Nobile CJ, Mitchell AP (2005) Regulation of cell-surface genes and biofilm formation by the C. albicans transcription factor Bcr1p. Curr Biol 15: 1150-1155.

7. Hausauer DL, Gerami-Nejad M, Kistler-Anderson C, Gale CA (2005) Hyphal guidance and invasive growth in Candida albicans require the Ras-like GTPase Rsr1p and its GTPase-activating protein Bud2p. Eukaryot Cell 4: 1273-1286.

8. Bruno VM, Mitchell AP (2005) Regulation of azole drug susceptibility by Candida albicans protein kinase CK2. Mol Microbiol 56: 559-573.

9. Chiang LY, Sheppard DC, Bruno VM, Mitchell AP, Edwards JE, Jr., et al. (2007) Candida albicans protein kinase CK2 governs virulence during oropharyngeal candidiasis. Cell Microbiol 9: 233-245.

10. Alem MA, Oteef MD, Flowers TH, Douglas LJ (2006) Production of tyrosol by Candida albicans biofilms and its role in quorum sensing and biofilm development. Eukaryot Cell 5: 1770-1779.

11. Braun BR, Johnson AD (2000) TUP1, CPH1 and EFG1 make independent contributions to filamentation in candida albicans. Genetics 155: 57-67.

12. Chamilos G, Lionakis MS, Lewis RE, Lopez-Ribot JL, Saville SP, et al. (2006) Drosophila melanogaster as a facile model for large-scale studies of virulence mechanisms and antifungal drug efficacy in Candida species. J Infect Dis 193: 1014-1022.

13. Lo HJ, Kohler JR, DiDomenico B, Loebenberg D, Cacciapuoti A, et al. (1997) Nonfilamentous C. albicans mutants are avirulent. Cell 90: 939-949.

14. Magee BB, Legrand M, Alarco AM, Raymond M, Magee PT (2002) Many of the genes required for mating in Saccharomyces cerevisiae are also required for mating in Candida albicans. Mol Microbiol 46: 1345-1351.

15. Chamilos G, Nobile CJ, Bruno VM, Lewis RE, Mitchell AP, et al. (2009) Candida albicans Cas5, a regulator of cell wall integrity, is required for virulence in murine and toll mutant fly models. J Infect Dis 200: 152-157.

16. Lane S, Zhou S, Pan T, Dai Q, Liu H (2001) The basic helix-loop-helix transcription factor Cph2 regulates hyphal development in Candida albicans partly via TEC1. Mol Cell Biol 21: 6418-6428.

17. Liu H (2001) Transcriptional control of dimorphism in Candida albicans. Curr Opin Microbiol 4: 728-735.

18. Giusani AD, Vinces M, Kumamoto CA (2002) Invasive filamentous growth of Candida albicans is promoted by Czf1p-dependent relief of Efg1p-mediated repression. Genetics 160: 1749-1753.

19. Martinez-Lopez R, Monteoliva L, Diez-Orejas R, Nombela C, Gil C (2004) The GPI-anchored protein CaEcm33p is required for cell wall integrity, morphogenesis and virulence in Candida albicans. Microbiology 150: 3341-3354.

20. Park H, Myers CL, Sheppard DC, Phan QT, Sanchez AA, et al. (2005) Role of the fungal Ras-protein kinase A pathway in governing epithelial cell interactions during oropharyngeal candidiasis. Cell Microbiol 7: 499-510.

21. Stoldt VR, Sonneborn A, Leuker CE, Ernst JF (1997) Efg1p, an essential regulator of morphogenesis of the human pathogen Candida albicans, is a member of a conserved class of bHLH proteins regulating morphogenetic processes in fungi. Embo J 16: 1982-1991.

22. Fan J, Whiteway M, Shen SH (2005) Disruption of a gene encoding glycerol 3-phosphatase from Candida albicans impairs intracellular glycerol accumulation-mediated salt-tolerance. FEMS Microbiol Lett 245: 107-116.

23. Wang A, Raniga PP, Lane S, Lu Y, Liu H (2009) Hyphal chain formation in Candida albicans: Cdc28-Hgc1 phosphorylation of Efg1 represses cell separation genes. Mol Cell Biol 29: 4406-4416.

24. Zheng X, Wang Y, Wang Y (2004) Hgc1, a novel hypha-specific G1 cyclin-related protein regulates Candida albicans hyphal morphogenesis. Embo J 23: 1845-1856.

25. Cheng S, Clancy CJ, Checkley MA, Handfield M, Hillman JD, et al. (2003) Identification of Candida albicans genes induced during thrush offers insight into pathogenesis. Mol Microbiol 48: 1275-1288.

26. Staab JF, Bradway SD, Fidel PL, Sundstrom P (1999) Adhesive and mammalian transglutaminase substrate properties of Candida albicans Hwp1. Science 283: 1535-1538.

27. Bailey DA, Feldmann PJ, Bovey M, Gow NA, Brown AJ (1996) The Candida albicans HYR1 gene, which is activated in response to hyphal development, belongs to a gene family encoding yeast cell wall proteins. J Bacteriol 178: 5353-5360.

28. Zakikhany K, Naglik JR, Schmidt-Westhausen A, Holland G, Schaller M, et al. (2007) In vivo transcript profiling of Candida albicans identifies a gene essential for interepithelial dissemination. Cell Microbiol 9: 2938-2954.

29. Lorenz MC, Fink GR (2001) The glyoxylate cycle is required for fungal virulence. Nature 412: 83-86.

30. Diez-Orejas R, Molero G, Navarro-Garcia F, Pla J, Nombela C, et al. (1997) Reduced virulence of Candida albicans MKC1 mutants: a role for mitogen-activated protein kinase in pathogenesis. Infect Immun 65: 833-837.

31. Kumamoto CA (2005) A contact-activated kinase signals Candida albicans invasive growth and biofilm development. Proc Natl Acad Sci U S A 102: 5576-5581.

32. Navarro-Garcia F, Alonso-Monge R, Rico H, Pla J, Sentandreu R, et al. (1998) A role for the MAP kinase gene MKC1 in cell wall construction and morphological transitions in Candida albicans. Microbiology 144 ( Pt 2): 411-424.

33. Leidich SD, Ibrahim AS, Fu Y, Koul A, Jessup C, et al. (1998) Cloning and disruption of caPLB1, a phospholipase B gene involved in the pathogenicity of Candida albicans. J Biol Chem 273: 26078-26086.

34. Prill SK, Klinkert B, Timpel C, Gale CA, Schroppel K, et al. (2005) PMT family of Candida albicans: five protein mannosyltransferase isoforms affect growth, morphogenesis and antifungal resistance. Mol Microbiol 55: 546-560.

35. Rouabhia M, Schaller M, Corbucci C, Vecchiarelli A, Prill SK, et al. (2005) Virulence of the fungal pathogen Candida albicans requires the five isoforms of protein mannosyltransferases. Infect Immun 73: 4571-4580.

36. Feng Q, Summers E, Guo B, Fink G (1999) Ras signaling is required for serum-induced hyphal differentiation in Candida albicans. J Bacteriol 181: 6339-6346.

37. Leberer E, Harcus D, Dignard D, Johnson L, Ushinsky S, et al. (2001) Ras links cellular morphogenesis to virulence by regulation of the MAP kinase and cAMP signalling pathways in the pathogenic fungus Candida albicans. Mol Microbiol 42: 673-687.

38. Davis D, Edwards JE, Jr., Mitchell AP, Ibrahim AS (2000) Candida albicans RIM101 pH response pathway is required for host-pathogen interactions. Infect Immun 68: 5953-5959.

39. Davis D, Wilson RB, Mitchell AP (2000) RIM101-dependent and-independent pathways govern pH responses in Candida albicans. Mol Cell Biol 20: 971-978.

40. Nobile CJ, Solis N, Myers CL, Fay AJ, Deneault JS, et al. (2008) Candida albicans transcription factor Rim101 mediates pathogenic interactions through cell wall functions. Cell Microbiol 10: 2180-2196.

41. Martchenko M, Alarco AM, Harcus D, Whiteway M (2004) Superoxide dismutases in Candida albicans: transcriptional regulation and functional characterization of the hyphal-induced SOD5 gene. Mol Biol Cell 15: 456-467.

42. Schweizer A, Rupp S, Taylor BN, Rollinghoff M, Schroppel K (2000) The TEA/ATTS transcription factor CaTec1p regulates hyphal development and virulence in Candida albicans. Mol Microbiol 38: 435-445.

43. Staib P, Binder A, Kretschmar M, Nichterlein T, Schroppel K, et al. (2004) Tec1p-independent activation of a hypha-associated Candida albicans virulence gene during infection. Infect Immun 72: 2386-2389.

44. Bockmuhl DP, Krishnamurthy S, Gerads M, Sonneborn A, Ernst JF (2001) Distinct and redundant roles of the two protein kinase A isoforms Tpk1p and Tpk2p in morphogenesis and growth of Candida albicans. Mol Microbiol 42: 1243-1257.

45. Sonneborn A, Bockmuhl DP, Gerads M, Kurpanek K, Sanglard D, et al. (2000) Protein kinase A encoded by TPK2 regulates dimorphism of Candida albicans. Mol Microbiol 35: 386-396.

46. Tebarth B, Doedt T, Krishnamurthy S, Weide M, Monterola F, et al. (2003) Adaptation of the Efg1p morphogenetic pathway in Candida albicans by negative autoregulation and PKA-dependent repression of the EFG1 gene. J Mol Biol 329: 949-962.

47. Braun BR, Head WS, Wang MX, Johnson AD (2000) Identification and characterization of TUP1-regulated genes in Candida albicans. Genetics 156: 31-44.

48. Murad AM, Leng P, Straffon M, Wishart J, Macaskill S, et al. (2001) NRG1 represses yeast-hypha morphogenesis and hypha-specific gene expression in Candida albicans. Embo J 20: 4742-4752.

49. Phan QT, Belanger PH, Filler SG (2000) Role of hyphal formation in interactions of Candida albicans with endothelial cells. Infect Immun 68: 3485-3490.

50. Palmer GE, Cashmore A, Sturtevant J (2003) Candida albicans VPS11 is required for vacuole biogenesis and germ tube formation. Eukaryot Cell 2: 411-421.

51. Palmer GE, Kelly MN, Sturtevant JE (2005) The Candida albicans vacuole is required for differentiation and efficient macrophage killing. Eukaryot Cell 4: 1677-1686.

52. Hromatka BS, Noble SM, Johnson AD (2005) Transcriptional response of Candida albicans to nitric oxide and the role of the YHB1 gene in nitrosative stress and virulence. Mol Biol Cell 16: 4814-4826.
