# Supplementary material for: From Attachment to Damage: Defined Genes of Candida albicans Mediate Adhesion, Invasion and Damage during Interaction with Oral Epithelial Cells
Source: PLoS One. 2011 Feb 23;6(2):e17046. doi: 10.1371/journal.pone.0017046 (PMC3044159; doi:10.1371/journal.pone.0017046)
Supplement: Table S2 — Morphology of C. albicans wild type and mutant strains (hyphal, pseudohyphal and yeast cells formation in %) and length of the formed filaments following 3 h incubation on epithelial monolayers. (DOC) [file pone.0017046.s002.doc]

**Table S2. Morphology of *C. albicans* wild type and mutant strains (hyphal, pseudohyphal and yeast cells formation in %) and length of the formed filaments following 3 h incubation on epithelial monolayers.**

| **strain** | **hyphae** | **pseudohyphae** | **yeast** | **filament length** | **SD** |
| --- | --- | --- | --- | --- | --- |
| SC5314 | 98.5 | <1 | <1 | 50.9 | 7.9 |
| BWP17+CIp30 | 98.5 | <1 | <1 | 50.6 | 7.6 |
| CAI-4 | 98.5 | <1 | <1 | 52.6 | 7.3 |
| RM1000 | 98.5 | <1 | <1 | 44.8 | 3.6 |
| *als3*Δ | 93.5 | 1.5 | 5 | 32.7 | 10.8 |
| *bcr1*Δ | 82 | 17.5 | 0.5 | 39.1 | 8.8 |
| *bud2*Δ | 4 | 95 | 1 | 40.1 | 11.5 |
| *cka2*Δ | 4 | 95 | 1 | 29.8 | 3.7 |
| *cph1*Δ | 95.4 | 4.4 | <1 | 47.8 | 9.7 |
| *cph2*Δ | 98 | 1 | 1 | 53.9 | 14.3 |
| *czf1*Δ | 84.7 | 15.3 | <1 | 30.4 | 5.4 |
| *ecm33*Δ | 38 | 34 | 18 | 27.2 | 12.4 |
| *ipf946Δ* (*eed1*Δ) | 83 | 11 | 6 | 43.2 | 6.7 |
| *efg1*Δ | <1 | 8 | 92 | 8.4 | 0.5 |
| *gpd2*Δ | 98.5 | <1 | <1 | 47.1 | 5.0 |
| *gpp1*Δ | 89 | 11 | <1 | 44.0 | 9.8 |
| *hgc1*Δ | 8 | 53 | 38 | 27.2 | 6.4 |
| *hwp1*Δ | 94 | 6 | <1 | 41.2 | 14.5 |
| *hyr1*Δ | 84 | 16 | <1 | 40.4 | 10.4 |
| *icl1* Δ | 43 | 57 | <1 | 45.2 | 6.8 |
| *mkc1*Δ | 98 | 2 | <1 | 50.6 | 8.0 |
| *plb1*Δ | 98 | 1 | 1 | 46.4 | 9.0 |
| *pmt2*Δ/*PMT2* | 78 | 18 | 4 | 31.3 | 10.5 |
| *ras1*Δ | <1 | <1 | > 99 | 0.1 | 0.4 |
| *rim101*Δ | 19 | 71 | 10 | 15.8 | 5.2 |
| *rsr1*Δ | 3 | 84 | 13 | 36.8 | 8.5 |
| *sod5* Δ | 98.5 | <1 | <1 | 48.4 | 1.3 |
| *tec1*Δ | 3 | 67 | 30 | 25.0 | 6.1 |
| *tpk1*Δ | 68 | 28 | 4 | 30.9 | 14.6 |
| *tpk2*Δ | 25 | 65 | 10 | 17.2 | 10.1 |
| *tup1*Δ | <1 | > 99 | <1 | / | / |
| *vps11* Δ | 7 | 56 | 37 | 9.2 | 3.0 |
| *yhb1* Δ | 98.5 | <1 | <1 | 51.7 | 7.5 |
| *orf19.851∆* | 98.5 | <1 | <1 | 30.34 | 9.31 |
| *orf19.2833∆* | 98.5 | <1 | <1 | 51.29 | 0.51 |
| *orf19.3459∆* | 98.5 | <1 | <1 | 28.21 | 7.26 |
| *orf19.3600∆* | 98.5 | <1 | <1 | 33.28 | 8.75 |
| *orf19.6837∆* | 98.5 | <1 | <1 | 29.91 | 6.67 |
