# Supplementary material for: From Attachment to Damage: Defined Genes of Candida albicans Mediate Adhesion, Invasion and Damage during Interaction with Oral Epithelial Cells
Source: PLoS One. 2011 Feb 23;6(2):e17046. doi: 10.1371/journal.pone.0017046 (PMC3044159; doi:10.1371/journal.pone.0017046)
Supplement: Table S3 — Summarized phenotypes of C. albicans wild type and mutant strains during interaction with epithelial cells. Comparison of published data to adhesion, invasion and damage properties of all strains investigated in this study. (DOC) [file pone.0017046.s003.doc]

**Table S3.** **Summarized phenotypes of** ***C. albicans* wild type and mutant strains during interaction with epithelial cells.**

|  |  | **Interaction with epithelial cells - published data** | | | | **Interaction with epithelial cells - this study** | | | |
| --- | --- | --- | --- | --- | --- | --- | --- | --- | --- |
| **Group** | **Tested mutant strain** | **Adhesion** | **Invasion** | **Damage** | **Reference** | **Adhesion (60 min) [%WT / (absolute numbers)]** | **Invasion (180 min) [%WT]** | **Damage (24 h) [%WT]** | **Invasion enterocytes (180 min) [%WT]** |
| 1 | *cph1*Δ | n.d. | n.d. | n.d. | / | 84.3 ± 12.6 (1.89E+04) | 91.7 ± 11.6 | 76.0 ± 19.4207 | 63.9 ± 13.8 |
| 1 | *cph2*Δ | n.d. | n.d. | n.d. | / | 79.4 ± 14.0 (1.78E+04) | 75.2 ± 8.8 | 87.1 ± 8.2 | 96.3 ± 16.7 |
| 1 | *hyr1*Δ | n.d. | n.d. | n.d. | / | 81.7 ± 16.6 (1.83E+04) | 84.4 ± 13.1 | 96.4 ± 26.7 | 70.2 ± 18.6 |
| 1 | *plb1*Δ | similar to WT | n.d. | n.d. | [1] | 86.9 ± 21 (1.95E+04) | 90.0 ± 22.4 | 107.9 ± 21.5 | 69.8 ± 27.4 |
| 2 | *als3*Δ | reduced | reduced | reduced | [2] | 35.4 ± 15.2 (7.93E+03) | 45.6 ± 15.1 | 32.2 ± 17.7 | 54.1 ± 16.8 |
| 2 | *czf1*Δ | n.d. | n.d. | n.d. | / | 34.9 ± 15.8 (7.82E+03) | 46.6 ± 7.8 | 20.8 ± 8.2 | 21.7 ± 19.7 |
| 2 | *ecm33*Δ | reduced | reduced | reduced | [3] | 55.7 ± 18.3 (1.25E+04) | 38.8 ± 13 | 24.7 ± 5.9 | 52.0 ± 20.2 |
| 2 | *efg1*Δ | reduced | reduced | reduced | [4] | 54.3 ± 9.5 (1.22E+04) | 28.6 ± 14.5 | 11.7 ± 15.7 | 3.8 ± 1.3 |
| 2 | *hgc1*Δ | n.d. | n.d. | n.d. | / | 51.9 ± 5.6 (1.16E+04) | 59.2 ± | 21.7 ± | 43.0 ± 17.5 |
| 2 | *ras1*Δ | n.d. | n.d. | n.d. | / | 1.0 ± 0.2 (2.31E+02) | 4.4 ± 2 | 14.6 ± 4.8 | 0.0 ± 0.7 |
| 2 | *rim101*Δ | increased* | reduced* | reduced | [5] | 20.4 ± 7.3 (4.58E+03) | 43.9 ± 10.5 | 23.3 ± 18.8 | 6.4 ± 7.8 |
| 2 | *tec1*Δ | n.d. | n.d. | n.d. | / | 5.3 ± 1.3 (1.19E+03) | 21.8 ± 11.3 | 13.8 ± 8.6 | 4.1 ± 3.5 |
| 2 | *tpk1*Δ | similar to WT | similar to WT | similar to WT | [4] | 4.8 ± 2.3 (1.07E+03 | 67.6 ± 13.3 | -2.5 ± 10.1 | 0.0 ± 4.6 |
| 2 | *tpk2*Δ | reduced | similar to WT | reduced | [4] | 2.1 ± 1.2 (4.69E+02) | 44.9 ± 4.3 | 6.9 ± 3.9 | 0.0 ± 3.0 |
| 2 | *tup1*Δ | reduced | n.d. | reduced | [6] | n.d. | 27.9 ± 18 | 2.2 ± 7.3 | 0.0 ± 0.5 |
| 2 | *vps11* Δ | n.d. | n.d. | n.d. | / | 2.7 ± 0.2 (5.95E+02) | 57.7 ± 12.3 | 6.5 ± 9.2 | 8.7 ± 9.4 |
| 3 | *pmt2*Δ/*PMT2* | n.d. | n.d. | n.d. | / | 98.7 ± 19 (2.21E+04) | 69.6 ± 6.2 | 16.4 ± 12 | 29.9 ± 6.5 |
| 4 | *ipf946Δ* (*eed1*Δ) | n.d. | n.d. | reduced | [7] | 83.2 ± 20.1 (1.86E+04) | 90.6 ± 6.2 | 7.8 ± 5.3 | 73.0 ± 17.7 |
| 4 | *gpd2*Δ | n.d. | n.d. | n.d. | / | 79.0 ± 17.3 (1.77E+04) | 82.1 ± 17 | 68.1 ± 20.9 | 61.2 ± 17.5 |
| 4 | *gpp1*Δ | n.d. | n.d. | n.d. | / | 76.6 ± 16.4 (1.72E+04) | 79.2 ± 16.5 | 51.0 ± 14.2 | 58.2 ± 14.7 |
| 4 | *mkc1*Δ | n.d. | n.d. | n.d. | / | 80.9 ± 20 (1.81E+04) | 87.2 ± 7.9 | 63.5 ± 12.7 | 103.3 ± 6.5 |
| 5 | *bcr1*Δ | n.d. | n.d. | n.d. | / | 16.3 ± 5.8 (3.64E+03) | 73.9 ± 16.4 | 62.3 ± 14.4 | 54.1 ± 2.6 |
| 5 | *bud2*Δ | n.d. | reduced | reduced | [8] | 6.6 ± 2.3 (1.48E+03) | 91.7 ± 21.6 | 26.5 ± 10.3 | 47.9 ± 11.1 |
| 5 | *cka2*Δ | reduced | reduced | reduced | [9] | 52.6 ± 14.9 (1.18E+04) | 82.8 ± 16.1 | 58.4 ± 6.8 | 28.7 ± 16.4 |
| 5 | *hwp1*Δ | reduced | n.d. | n.d. | [10] | 28.4 ± 12.6 (6.36E+03) | 76.8 ± 22.8 | 50.1 ± 17.8 | 43.4 ± 11.8 |
| 5 | *rsr1*Δ | n.d. | reduced | reduced | [8] | 16.7 ± 14.5 (3.74E+03) | 78.9 ± 4.6 | 3.0 ± 2.1 | 42.4 ± 19.9 |
| * | *icl1*Δ | n.d. | n.d. | n.d. | / | 69.5 ± 6.7 (1.56E+04) | 87.0 ± 8.3 | 66.5 ± 18.5 | 62.0 ± 18 |
| * | *PGA34∆ (orf19.2833∆)* | n.d. | n.d. | n.d. | / | 93 ± 25.1 (2.08E+04) | 104.5 ± 17.5 | 58 ± 11.6 | n.d. |
| * | *sod5*Δ | n.d. | n.d. | n.d. | / | 97.1 ± 24.6 (2.18E+04) | 92.7 ± 18.1 | 97.6 ± 5.5 | n.d. |
| * | *yhb1*Δ | n.d. | n.d. | n.d. | / | 95.2 ± 13.3 (2.13E+04) | 98.6 ± 14.1 | 87.1 ± 7.8 | n.d. |
| WT | SC5314 (WT) |  |  |  | [11,12] | 22 % (2.24E+04) | 70 % | 41 %^#^ | 24.2 % |
| WT | BWP17 |  |  |  | [13] | similar to SC5314 | similar to SC5314 | similar to SC5314 | similar to SC5314 |
| WT | CAI-4 |  |  |  | [14] | similar to SC5314 | similar to SC5314 | similar to SC5314 | similar to SC5314 |
| WT | RM1000 |  |  |  | [15] | similar to SC5314 | similar to SC5314 | similar to SC5314 | similar to SC5314 |
| * | *orf19.851∆* | n.d. | n.d. | n.d. | / | n.d. | 82.5 ± 0.7 | 116.43 ± 10.3 | n.d. |
| * | *orf19.3459∆* | n.d. | n.d. | n.d. | / | n.d. | 77 ± 1.4 | 106.82 ± 11.9 | n.d. |
| * | *orf19.3600∆* | n.d. | n.d. | n.d. | / | n.d. | 76.5 ± 3.5 | 108.7 ± 13.73 | n.d. |
| * | *orf19.6837∆* | n.d. | n.d. | n.d. | / | n.d. | 80 ± 2.8 | 120.81 ± 32.1 | n.d. |

Comparison of published data to adhesion, invasion and damage properties of all strains investigated in this study. Green: similar to wild type or moderately reduced (but not significantly reduced); red: significant different to wild type (*p* < 0.05); * *nrg1*Δ/*rim101*Δ+pRIM101; ^#^ compared to 100 % lysis (triton); n.d. = not determined.

1. Leidich SD, Ibrahim AS, Fu Y, Koul A, Jessup C, et al. (1998) Cloning and disruption of ca*PLB1*, a phospholipase B gene involved in the pathogenicity of *Candida albicans*. J Biol Chem 273: 26078-26086.

2. Phan QT, Myers CL, Fu Y, Sheppard DC, Yeaman MR, et al. (2007) Als3 is a *Candida albicans* invasin that binds to cadherins and induces endocytosis by host cells. PLoS Biol 5: e64.

3. Martinez-Lopez R, Park H, Myers CL, Gil C, Filler SG (2006) *Candida albicans* Ecm33p is important for normal cell wall architecture and interactions with host cells. Eukaryot Cell 5: 140-147.

4. Park H, Myers CL, Sheppard DC, Phan QT, Sanchez AA, et al. (2005) Role of the fungal Ras-protein kinase A pathway in governing epithelial cell interactions during oropharyngeal candidiasis. Cell Microbiol 7: 499-510.

5. Nobile CJ, Solis N, Myers CL, Fay AJ, Deneault JS, et al. (2008) *Candida albicans* transcription factor Rim101 mediates pathogenic interactions through cell wall functions. Cell Microbiol 10: 2180-2196.

6. Villar CC, Kashleva H, Dongari-Bagtzoglou A (2004) Role of *Candida albicans* polymorphism in interactions with oral epithelial cells. Oral Microbiol Immunol 19: 262-269.

7. Zakikhany K, Thewes S, Wilson D, Martin R, Albrecht A, et al. (2008) From attachment to invasion: infection associated genes of *Candida albicans*. Nippon Ishinkin Gakkai Zasshi 49: 245-251.

8. Brand A, Vacharaksa A, Bendel C, Norton J, Haynes P, et al. (2008) An internal polarity landmark is important for externally induced hyphal behaviors in *Candida albicans*. Eukaryot Cell 7: 712-720.

9. Chiang LY, Sheppard DC, Bruno VM, Mitchell AP, Edwards JE, Jr., et al. (2007) *Candida albicans* protein kinase CK2 governs virulence during oropharyngeal candidiasis. Cell Microbiol 9: 233-245.

10. Staab JF, Bradway SD, Fidel PL, Sundstrom P (1999) Adhesive and mammalian transglutaminase substrate properties of *Candida albicans* Hwp1. Science 283: 1535-1538.

11. Fonzi WA, Irwin MY (1993) Isogenic strain construction and gene mapping in *Candida albicans*. Genetics 134: 717-728.

12. Gillum AM, Tsay EY, Kirsch DR (1984) Isolation of the *Candida albicans* gene for orotidine-5'-phosphate decarboxylase by complementation of *S. cerevisiae* ura3 and *E. coli* pyrF mutations. Mol Gen Genet 198: 179-182.

13. Wilson RB, Davis D, Mitchell AP (1999) Rapid hypothesis testing with *Candida albicans* through gene disruption with short homology regions. J Bacteriol 181: 1868-1874.

14. Brand A, MacCallum DM, Brown AJ, Gow NA, Odds FC (2004) Ectopic expression of *URA3* can influence the virulence phenotypes and proteome of *Candida albicans* but can be overcome by targeted reintegration of *URA3* at the RPS10 locus. Eukaryot Cell 3: 900-909.

15. Negredo A, Monteoliva L, Gil C, Pla J, Nombela C (1997) Cloning, analysis and one-step disruption of the *ARG5,6* gene of *Candida albicans*. Microbiology 143 ( Pt 2): 297-302.
