# Supplementary material for: From Attachment to Damage: Defined Genes of Candida albicans Mediate Adhesion, Invasion and Damage during Interaction with Oral Epithelial Cells
Source: PLoS One. 2011 Feb 23;6(2):e17046. doi: 10.1371/journal.pone.0017046 (PMC3044159; doi:10.1371/journal.pone.0017046)
Supplement: Table S6 — List of the genotypes of C. albicans strains used in this study. (DOC) [file pone.0017046.s006.doc]

**Table S6. List of *C. albicans* strains used in this study and their genotypes.**

| **Strain** | **Genotype** | **Reference** |
| --- | --- | --- |
| ***als3*Δ** | *BWP17 als3Δ::ARG4/als3Δ::HIS1* | [1] |
| ***bcr1*Δ** | *BWP17 bcr1Δ::ARG1/bcr1Δ::URA3* | [2] |
| ***bud2*Δ** | *BWP17 bud2Δ::ARG4/bud2:Δ:HIS1* | [3] |
| ***cka2*Δ** | *BWP17 cka2Δ::HIS1/cka2Δ::ARG4* | [4] |
| ***cph1*Δ** | *CAI-4 cph1Δ::hisG/cph1Δ::hisG* | [5] |
| ***cph2*Δ** | *BWP17 cph2Δ::ARG4/cph2Δ::HIS1 ADE2/ade2::URA3* | [6] |
| ***czf1*Δ** | *CAI-4 czf1Δ::hisG/czf1Δ::hisG ade2::pMal2-URA3* | [7] |
| ***ecm33*Δ** | *CAI-4 ecm33Δ::hisG/ecm33Δ::hisG* | [8] |
| ***efg1*Δ** | *CAI-4 efg1Δ::hisG/efg1Δ::hisG RPS10::pCIP10* | [6] |
| ***gpd2*Δ** | *BWP17 gpd2Δ::ARG4/gpd2Δ::HIS1 RPS10::pCIP10* | this study |
| ***gpp1*Δ** | *BWP17 gpp1Δ::ARG4/gpp1Δ::HIS1 RPS10::pCIP10* | this study |
| ***hgc1*Δ** | *BWP17 hgc1Δ::ARG4/hgc1Δ::HIS1 RPS10::pCIP10* | [9] |
| ***hwp1*Δ** | *CAI-4 hwp1Δ::hisG/hwp1Δ::hisG* | [10] |
| ***hyr1*Δ** | *CAI-4 hyr1Δ::hisG/hyr1Δ::hisG* | [11] |
| ***eed1*Δ** | *BWP17 eed1Δ::HIS1/eed1Δ::ARG4 RPS10::pCIP10* | [12] |
| ***eed1Δ + EED1*** | *Revertant strain eed1Δ/EED1::EED1 RPS10::pCIP30* | [12] |
| ***icl1*Δ** | *CAI-4 icl1Δ::hisG/icl1Δ::hisG* | [13] |
| ***mkc1*Δ** | *CAI-4 mkc1Δ::hisG/mkc1Δ::hisG mkc1::pCK70* | [14] |
| ***plb1*Δ** | *CAI-4 plb1Δ::hisG/plb1Δ::hisG* | [15] |
| ***pmt2*Δ/*PMT2*** | *CAI-4 pmt2Δ::hisG/PMT2* | [16] |
| ***ras1*Δ** | *CAI-4 ras1Δ::hisG/ras1Δ::hph* | [17] |
| ***rim101*Δ** | *CAI-4 prr2Δ::hisG/prr2Δ::hisG* | [18] |
| ***rsr1*Δ** | *BWP17 rsr1Δ::ARG4/rsr1Δ::HIS1* | [3] |
| ***sod5*Δ** | *CAI-4 sod5Δ::hisG/sod5Δ::hisG RPS10::pCIP10* | [19] |
| ***tec1*Δ** | *CAI-4 tec1Δ::hisG/tec1Δ::hisG* | [20] |
| ***tpk1*Δ** | *CAI-4 tpk1Δ::hisG/tpk1Δ::hisG* | [21] |
| ***tpk2*Δ** | *CAI-4 tpk2Δ::hisG/tpk2Δ::hisG* | [21] |
| ***tup1*Δ** | *CAI-4 tup1Δ:hisG/tup1Δ:hisG* | [22] |
| ***vps11* Δ** | *BWP17 vps11Δ::ARG4/vps11Δ::URA3* | [23] |
| ***yhb1* Δ** | *RM1000 yhb1Δ::hisG/yhb1Δ::HIS1* | [24] |
| ***orf19.851∆*** | *BWP17 orf19.851Δ::ARG4/orf19.851Δ::HIS1 RPS10::pCIP10* | this study |
| ***orf19.2833∆*** | *BWP17 orf19.2833Δ::ARG4/orf19.2833Δ::HIS1 RPS10::pCIP10* | this study |
| ***orf19.3459∆*** | *BWP17 orf19.3459Δ::ARG4/orf19.3459Δ::HIS1 RPS10::pCIP10* | this study |
| ***orf19.3600∆*** | *BWP17 orf19.3600Δ::ARG4/orf19.3600::HIS1Δ RPS10::pCIP10* | this study |
| ***orf19.6837∆*** | *BWP17 orf19.6837Δ::ARG4/orf19.6837Δ::HIS1 RPS10::pCIP10* | this study |
| **SC5314** | Isogenic wild type | [25] |
| **CAI-4** | *ura3::imm434/ura3::imm434 iro1/iro1::imm434/ RPS10::pCIP10* | [19] |
| **BWP17** | *ura3::imm434/ura3::imm434 iro1/iro1::imm434 his1::hisG/his1::hisG arg4/arg4 RPS10::pCIP30* | [26] |
| **RM1000** | *ura3::imm434/ura3::imm434 iro1/iro1::imm434 his1::hisG/his1::hisG* | [27] |

1. Phan QT, Myers CL, Fu Y, Sheppard DC, Yeaman MR, et al. (2007) Als3 is a *Candida albicans* invasin that binds to cadherins and induces endocytosis by host cells. PLoS Biol 5: e64.

2. Nobile CJ, Andes DR, Nett JE, Smith FJ, Yue F, et al. (2006) Critical role of Bcr1-dependent adhesins in C. albicans biofilm formation in vitro and in vivo. PLoS Pathog 2: e63.

3. Brand A, Vacharaksa A, Bendel C, Norton J, Haynes P, et al. (2008) An internal polarity landmark is important for externally induced hyphal behaviors in *Candida albicans*. Eukaryot Cell 7: 712-720.

4. Chiang LY, Sheppard DC, Bruno VM, Mitchell AP, Edwards JE, Jr., et al. (2007) *Candida albicans* protein kinase CK2 governs virulence during oropharyngeal candidiasis. Cell Microbiol 9: 233-245.

5. Liu H, Kohler J, Fink GR (1994) Suppression of hyphal formation in *Candida albicans* by mutation of a *STE12* homolog. Science 266: 1723-1726.

6. Lane S, Zhou S, Pan T, Dai Q, Liu H (2001) The basic helix-loop-helix transcription factor Cph2 regulates hyphal development in *Candida albicans* partly via *TEC1*. Mol Cell Biol 21: 6418-6428.

7. Brown DH, Jr., Giusani AD, Chen X, Kumamoto CA (1999) Filamentous growth of *Candida albicans* in response to physical environmental cues and its regulation by the unique *CZF1* gene. Mol Microbiol 34: 651-662.

8. Martinez-Lopez R, Park H, Myers CL, Gil C, Filler SG (2006) *Candida albicans* Ecm33p is important for normal cell wall architecture and interactions with host cells. Eukaryot Cell 5: 140-147.

9. Zheng X, Wang Y, Wang Y (2004) Hgc1, a novel hypha-specific G1 cyclin-related protein regulates *Candida albicans* hyphal morphogenesis. Embo J 23: 1845-1856.

10. Staab JF, Bradway SD, Fidel PL, Sundstrom P (1999) Adhesive and mammalian transglutaminase substrate properties of *Candida albicans* Hwp1. Science 283: 1535-1538.

11. Bailey DA, Feldmann PJ, Bovey M, Gow NA, Brown AJ (1996) The *Candida albicans* HYR1 gene, which is activated in response to hyphal development, belongs to a gene family encoding yeast cell wall proteins. J Bacteriol 178: 5353-5360.

12. Zakikhany K, Naglik JR, Schmidt-Westhausen A, Holland G, Schaller M, et al. (2007) In vivo transcript profiling of *Candida albicans* identifies a gene essential for interepithelial dissemination. Cell Microbiol 9: 2938-2954.

13. Lorenz MC, Fink GR (2001) The glyoxylate cycle is required for fungal virulence. Nature 412: 83-86.

14. Kumamoto CA (2005) A contact-activated kinase signals *Candida albicans* invasive growth and biofilm development. Proc Natl Acad Sci U S A 102: 5576-5581.

15. Ghannoum MA (1998) Extracellular phospholipases as universal virulence factor in pathogenic fungi. Nippon Ishinkin Gakkai Zasshi 39: 55-59.

16. Prill SK, Klinkert B, Timpel C, Gale CA, Schroppel K, et al. (2005) *PMT* family of *Candida albicans*: five protein mannosyltransferase isoforms affect growth, morphogenesis and antifungal resistance. Mol Microbiol 55: 546-560.

17. Feng Q, Summers E, Guo B, Fink G (1999) Ras signaling is required for serum-induced hyphal differentiation in *Candida albicans*. J Bacteriol 181: 6339-6346.

18. Ramon AM, Porta A, Fonzi WA (1999) Effect of environmental pH on morphological development of *Candida albicans* is mediated via the PacC-related transcription factor encoded by *PRR2*. J Bacteriol 181: 7524-7530.

19. Fradin C, De Groot P, MacCallum D, Schaller M, Klis F, et al. (2005) Granulocytes govern the transcriptional response, morphology and proliferation of *Candida albicans* in human blood. Mol Microbiol 56: 397-415.

20. Schweizer A, Rupp S, Taylor BN, Rollinghoff M, Schroppel K (2000) The TEA/ATTS transcription factor CaTec1p regulates hyphal development and virulence in *Candida albicans*. Mol Microbiol 38: 435-445.

21. Sonneborn A, Bockmuhl DP, Gerads M, Kurpanek K, Sanglard D, et al. (2000) Protein kinase A encoded by *TPK2* regulates dimorphism of *Candida albicans*. Mol Microbiol 35: 386-396.

22. Braun BR, Head WS, Wang MX, Johnson AD (2000) Identification and characterization of *TUP1*-regulated genes in *Candida albicans*. Genetics 156: 31-44.

23. Palmer GE, Cashmore A, Sturtevant J (2003) *Candida albicans* *VPS11* is required for vacuole biogenesis and germ tube formation. Eukaryot Cell 2: 411-421.

24. Hromatka BS, Noble SM, Johnson AD (2005) Transcriptional response of *Candida albicans* to nitric oxide and the role of the *YHB1* gene in nitrosative stress and virulence. Mol Biol Cell 16: 4814-4826.

25. Gillum AM, Tsay EY, Kirsch DR (1984) Isolation of the *Candida albicans* gene for orotidine-5'-phosphate decarboxylase by complementation of *S. cerevisiae* ura3 and *E. coli* pyrF mutations. Mol Gen Genet 198: 179-182.

26. Wilson RB, Davis D, Mitchell AP (1999) Rapid hypothesis testing with *Candida albicans* through gene disruption with short homology regions. J Bacteriol 181: 1868-1874.

27. Negredo A, Monteoliva L, Gil C, Pla J, Nombela C (1997) Cloning, analysis and one-step disruption of the *ARG5,6* gene of *Candida albicans*. Microbiology 143 ( Pt 2): 297-302.
